# Supplementary material for: A glyoxal-specific aldehyde signaling axis in Pseudomonas aeruginosa that influences quorum sensing and infection
Source: Nat Commun. 2025 Jul 18;16:6616. doi: 10.1038/s41467-025-61469-8 (PMC12274486; doi:10.1038/s41467-025-61469-8)
Supplement: Supplementary file 1 — Supplementary Information [file 41467_2025_61469_MOESM1_ESM.pdf]

Supplementary Table 1. Data collection and refinement statistics for ArqI crystal structures.

	Se-SAD	Native - modified	Native	R16A
**Wavelength of collection**	12662 eV	12398 eV	12398 eV	12398 eV
**Data collection**	SSRL 12-2	ALS 5.0.2	SSRL 12-2	SSRL 12-2
Space group	$P\ 2_1\ 2_1\ 2$	$C\ 2\ 2\ 2_1$	$P\ 2_1$	H 3
Cell dimensions				
a, *b*, *c* (Å)	111.86, 119.79, 57.52	109.0, 149.0, 120.2	61.4, 187.4, 60.4	62.8, 62.8, 120.4
α, β, γ (°)	90, 90, 90	90, 90, 90	90, 99.2, 90	90, 90, 120
Resolution (Å)	38.9-2.0 (2.1-2.0)[a]	49.6-2.0 (2.1-2.0)	39.2-2.1 (2.14-2.1)	31.4-1.5 (1.52-1.50)
R_{merge}[b]	0.204 (1.525)	0.15 (1.3)	0.19 (1.92)	0.05 (0.364)
$I / \sigma I$	11.4 (1.8)	13.5 (2.2)	8.7 (2.2)	16.3 (3.1)
Completeness (%)	99.2 (97.6)	100 (100)	99 (97.9)	99.6 (96.4)
Redundancy	13.4 (13.2)	13.4 (13.4)	7.0 (7.0)	5.7 (5.4)
**Refinement**				
Resolution (Å)	38.9-2.0 (2.1-2.0)	49.6-2.0 (2.1-2.0)	38.3-2.1 (2.2-2.1)	26.5-1.5 (1.6-1.5)
Total reflections	105039 (10305)	846662 (59361)	537963 (31853)	162474 (7293)
Unique reflections	52555 (5153)	63379 (4439)	76341 (4541)	28306 (1350)
R_{work} / R_{free}[c]	16.3 / 20.1	16.9 / 17.1	17.1 /20.8	17.6 / 20.5
Ramachandran favored (%)	97.8	98.8	98.4	97.8
Ramachandran outliers (%)	0	0.2	0	0
Subunits in ASU	6	6	12	2
No. atoms				
Protein	5011	5039	9707	1611
Ligands	129	58	570	73
Water	639	774	528	253
B-factors ($Å^2$)				
Protein	23.7	29.6	30.6	20.9
Ligands	39.6	40.3	45.5	36.1
Water	35.1	40.9	40.9	32.6
R.m.s. deviations				
Bond lengths (Å)	0.011	0.003	0.003	0.006
Bond angles (°)	1.16	0.64	0.86	0.92
PDB ID		8ECX	8EIF	8ECP

[a]. Values within parentheses refer to the highest resolution shell.

[b]. $R_{merge} = \sum\sum|I_{hkl} — I_{hkl}(j)\ |/\sum I_{hkl}$, where $I_{hkl}(j)$ is observed intensity and I_{hkl} is the final average value of intensity.

[c]. $R_{work} = \sum||F_{obs}| - |F_{calc}||/\sum|F_{obs}|$ and $R_{free} = \sum||F_{obs}| - |F_{calc}||/\sum|F_{obs}|$, where all reflections belong to a test set of 5% data randomly selected in Phenix.

Supplementary Figure 1

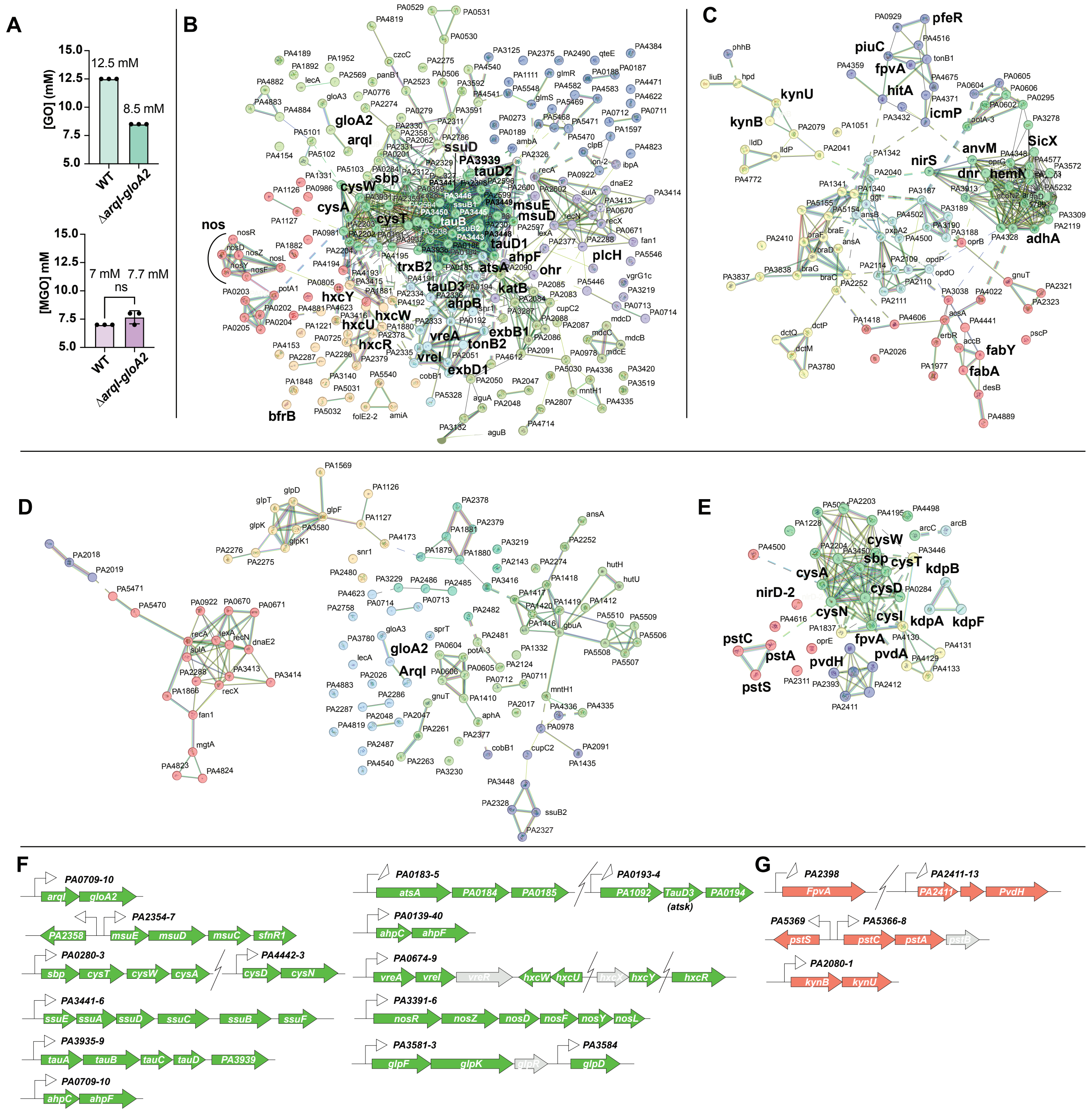

Supplementary Figure 1. **GO and MGO Minimal Inhibitory Concentration (MIC) and graphic output of STRING cluster analyses.** (**A**) MIC of GO and MGO treatment of the MPO1 wild-type versus Δ*arqI-gloA2* deletion strain as determined from three biological repeats. (**B**) STRING [1] output of GO treated 15-minute timepoint of upregulated and (**C**) downregulated genes. (**D**) STRING output of GO treated 1-hour timepoint upregulated and (**E**) downregulated genes. Some genes that are mentioned in the text are bolded and appear as a larger font size for better visualization. (**F**) Schematic representation of some selected operons / genes that were upregulated (green) and (**G**) downregulated (red) with GO treatment. Locus numbers for PAO1 genes are given above. Genes that appear as grey arrows in both *F* and *G* were not significantly changed with GO addition. Source data are provided as a Source Data file.

Supplementary Figure 2

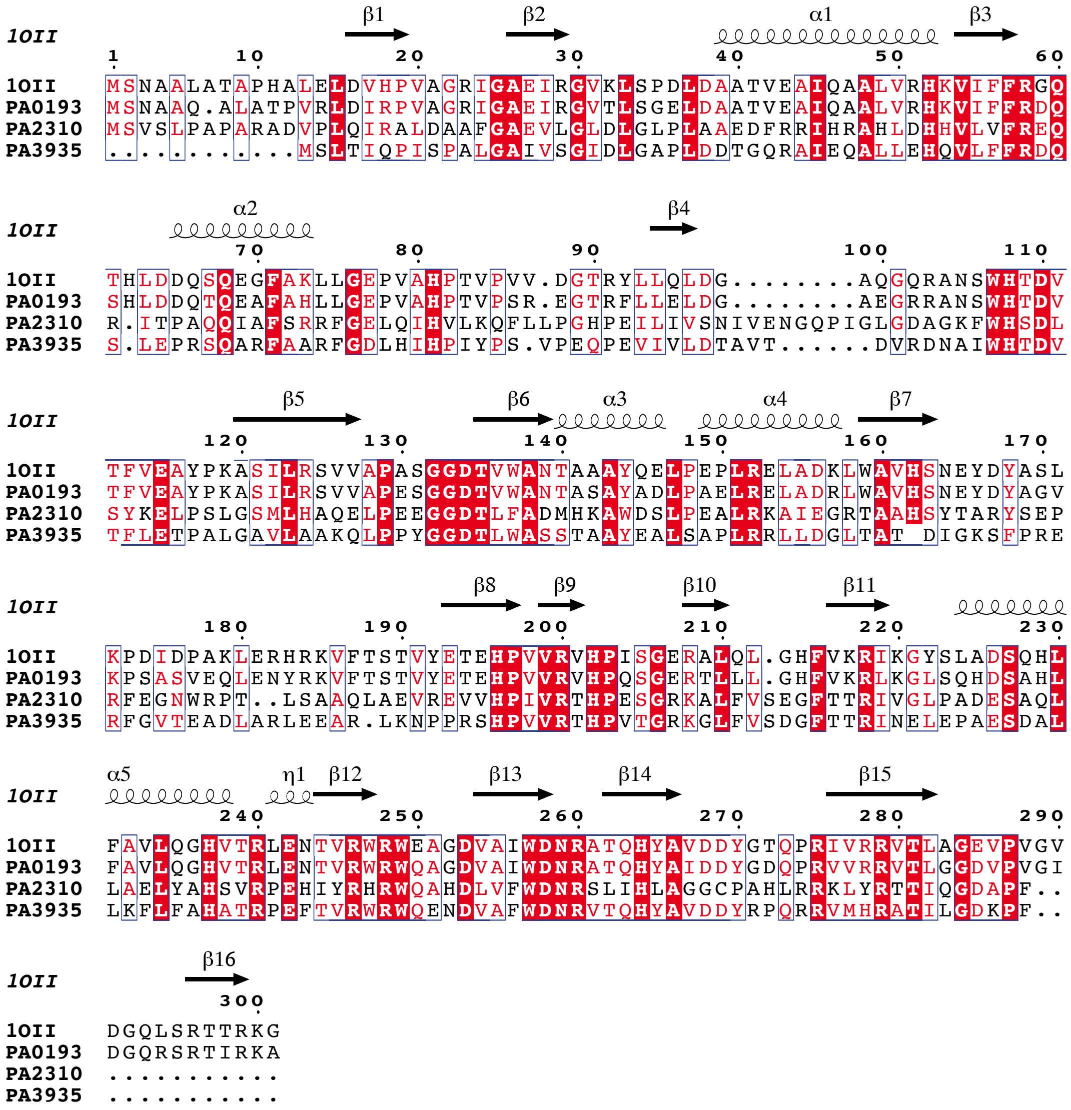

Supplementary Figure 2. **Alignment of TauD homologs**. *P. aeruginosa* contains three TauD homologs (PA3935, TauD1; PA2310, TauD2; TA0193, TauD3). Identical residues are boxed in red and similar residues have red letters. A closely related structure in the TauD superfamily from *Pseudomonas putida*, the alkylsulfatase AstK (PDB code: 1OII; [2]) was included to supply secondary structure labeling to the alignment. Clustal Omega [3] was used to generate the alignment and ESPript3 [4] was used to display the Clustal Omega output file.

Supplementary Figure 3

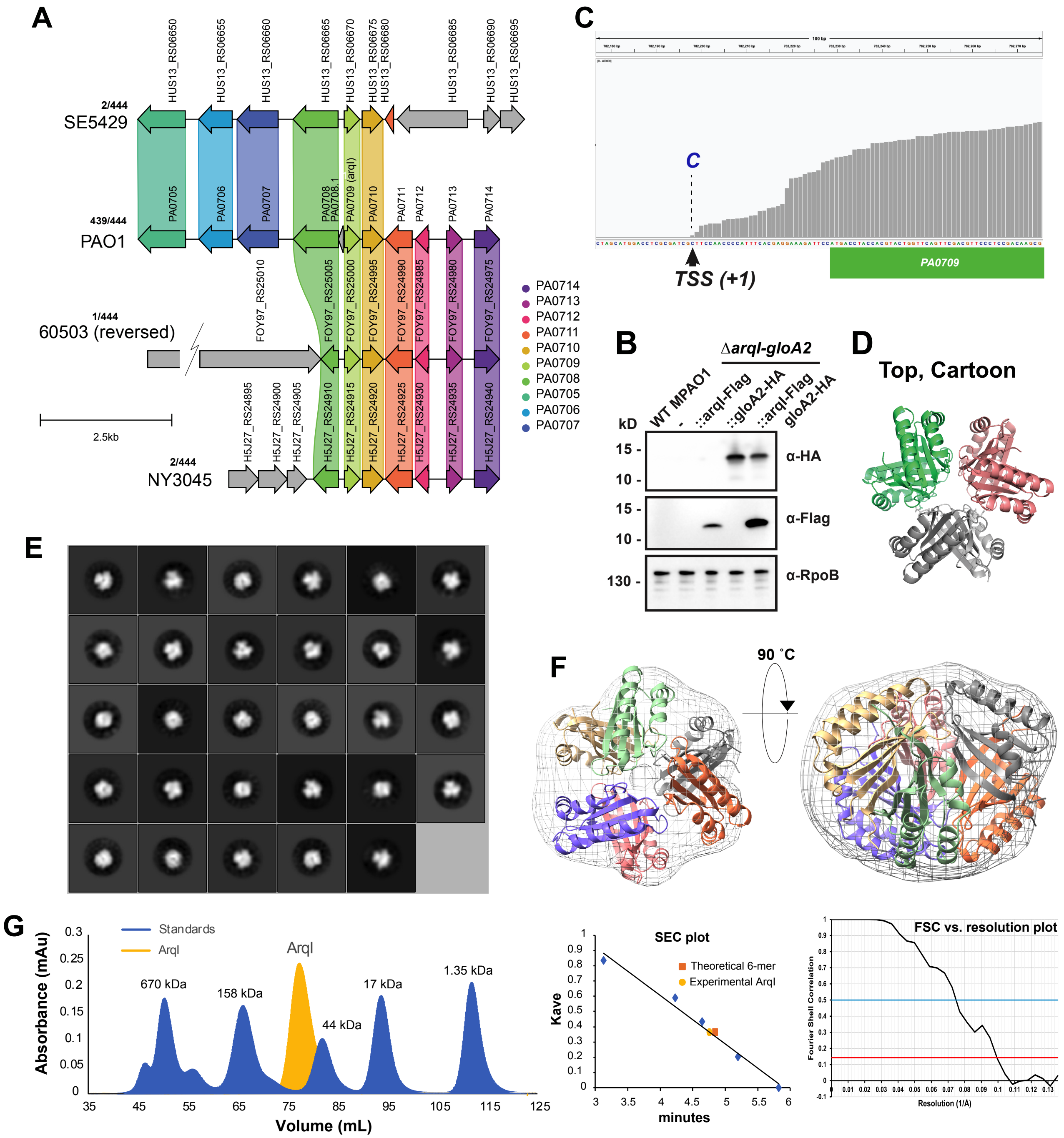

Supplementary Figure 3. The arqI-gloA operon, ArqI EM and its oligomeric state. (**A**) arqI-gloA2 and surrounding gene synteny among deposited P. aeruginosa genomes. The number of genomes with the SE5429, PAO1 and 60503 synteny is expressed as a fraction of the 444-genome total above the name. Locus numbers are given above the genes. (**B**) Western blot analysis of tagged FLAG-ArqI and GloA2-HA complements used in *Fig. 1C, D*. (**C**) Identification of a potential arqI-gloA2 operon transcriptional start site (+1) from RNA-seq data using Integrated Gene Viewer [5]. (D) Cartoon depiction of the ArqI hexamer wheel-like crystal structure. Each dimer is shown in a different color (salmon, green, and grey). (**E**) Representative negative stain EM 2D class averages of wild-type ArqI particles. (**F**) Above: The ArqI crystal structural model was fit as a rigid body into the negative stain EM volume. The diagrams show "top" (left) and “side” (right) views of the ArqI wheel fit into the volume. Every monomer is colored differently. Below: Fourier Shell Correlation (FSC) versus Resolution plot. The curve shows the FSC for the negative stain data. The red line corresponds to the 0.143 resolution criteria and the blue line to the 0.5 resolution criteria. (**G**) SEC graph (left) and plot (right). Source data are provided as a Source Data file.

Supplementary Figure 4

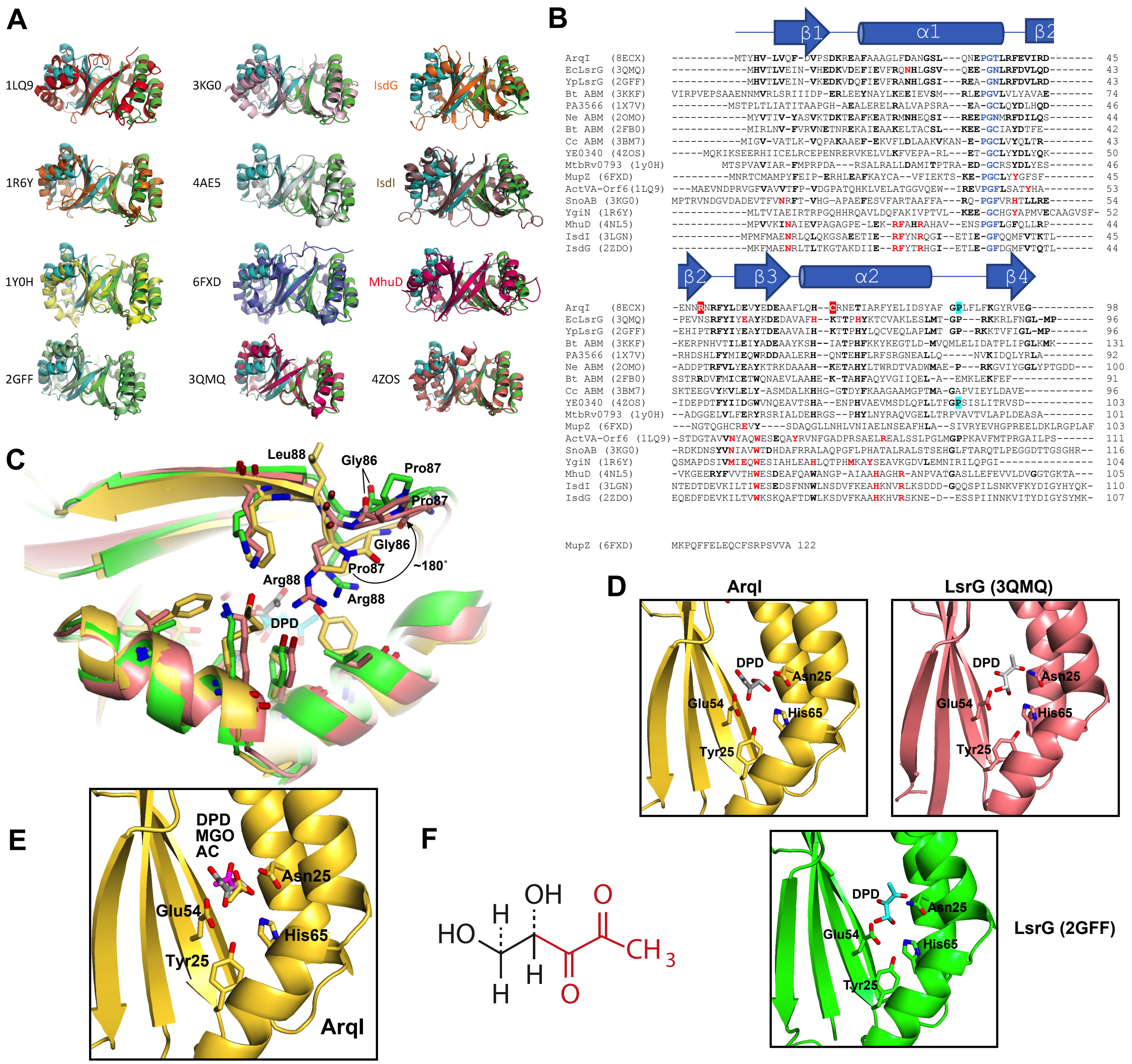

Supplementary Figure 4. **Structural and sequence comparison of ArqI dimer with structural homologs.** (**A**) Alignment of the ArqI dimeric structure (chains A and B, colored as above) with structural homologs: *Streptomyces coelicolor* ActVA-Orf6 (1LQ9, red), *E. coli* YgiN (1R6Y, orange), *Mycobacterium tuberculosis* Rv0793 (1Y0H, yellow), *Yersinia pestis* LsrG (2GFF, green), *Streptomyces nogalater* SnoaB (3KG0, pink), *Staphylococcus aureus* TRAP (4AE5, silver), *Pseudomonas fluorescens* MupZ (6FXD, purple), *E. coli* LsrG (3QMQ, magenta), *S. aureus* IsdG (2ZDO, orange), IsdI (3LGN, brown), *M. tuberculosis* MhuD (4NL5, magenta), and *Yersinia enterocolitica* YE0340 (4ZOS, salmon). (**B**) Alignment of ArqI sequence with structural homologs. Protein sequences are from *A*. Above the sequences is a cartoon representation of the ArqI secondary structure. Active site residues identified in the literature are colored red. A conserved PG (N/C/F) motif present in most of the homologs is colored blue. Modified residues in ArqI structures (Arg49 and Cys68) are boxed in red. The ArqI *cis*-proline is highlighted in teal. Residues conserved with ArqI across structures are bolded. (**C**) Overlay of the ArqI (in yellow), 2GFF and 3QMQ monomers emphasizing the Pro86/Gly87/Val88 ~180˚ rotation due to the *cis*-proline in ArqI. (**D**) ArqI (yellow; PDB code 8ECX, this work), *E. coli* LsrG (salmon; PDB code 3QMQ), and *Y. pestis* (green; PDB code 2GFF) modeled using HDOCK [6] with 4,5-Dihydroxy-2,3-pentanedione (DPD). (**E**) ArqI with DPD, methylglyoxal (MGO), and Acetate (AC) modeled with HDOCK [6]. (**F**) Structure of 4,5-Dihydroxy-2,3-pentanedione (DPD) with the methylglyoxal structure in red.

Supplementary Figure 5

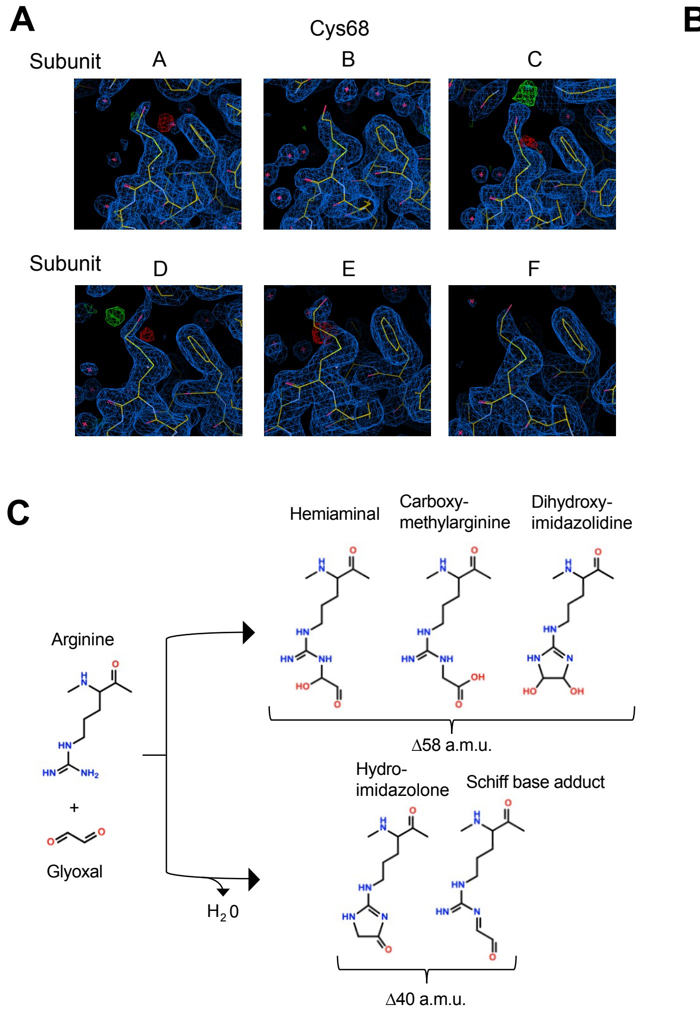

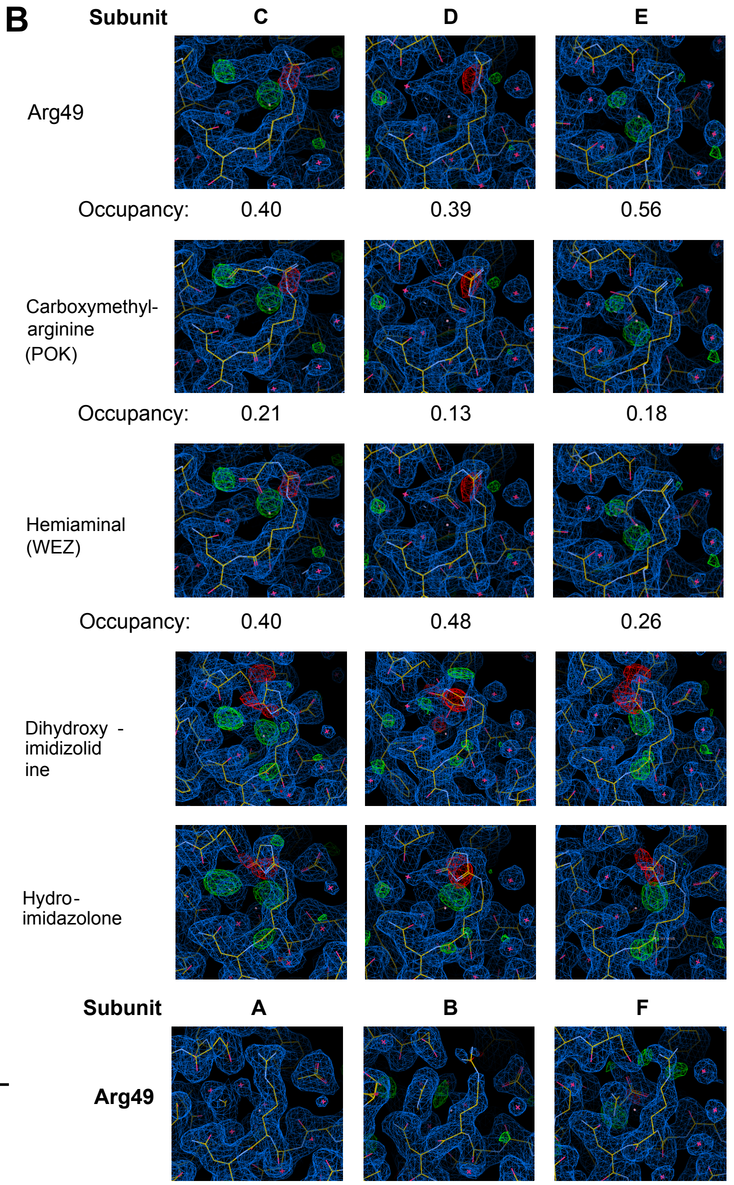

D

	Modification	Residue number	Peptide	Observed [1]	MR [2]	Calculated [3]	ppm [4]	M [5]	Score [6]	Expect [7]
Cys68	None	52-69	R.FYLDEVYEDEAAFLQH**C**R.N	750.01	2247	2247	1.01	0	62	4.40E- 06
	βME (+76)	52-69	R.FYLDEVYEDEAAFLQH**C**R.N	1162.5	2323	2323	-0.93	0	115	1.70E- 11
Arg49	None	39-49	L.RFEVIRDENNR.N	483.25	1447	1447	0.56	2	32	0.028
	Glyoxal (+58)	39-49	L.RFEVIRDENN**R**.N	502.59	1505	1505	9.16	2	26	0.0088

(1) Observed mass; (2) expected mass; (3) calculated mass; (4) parts per million; (5) missed trypsin cleavages; (6) ion score; (7) frequency the match would occur by chance. βME, β-mercaptoethanol. Cys68 and Arg49 residues are bolded and underlined.

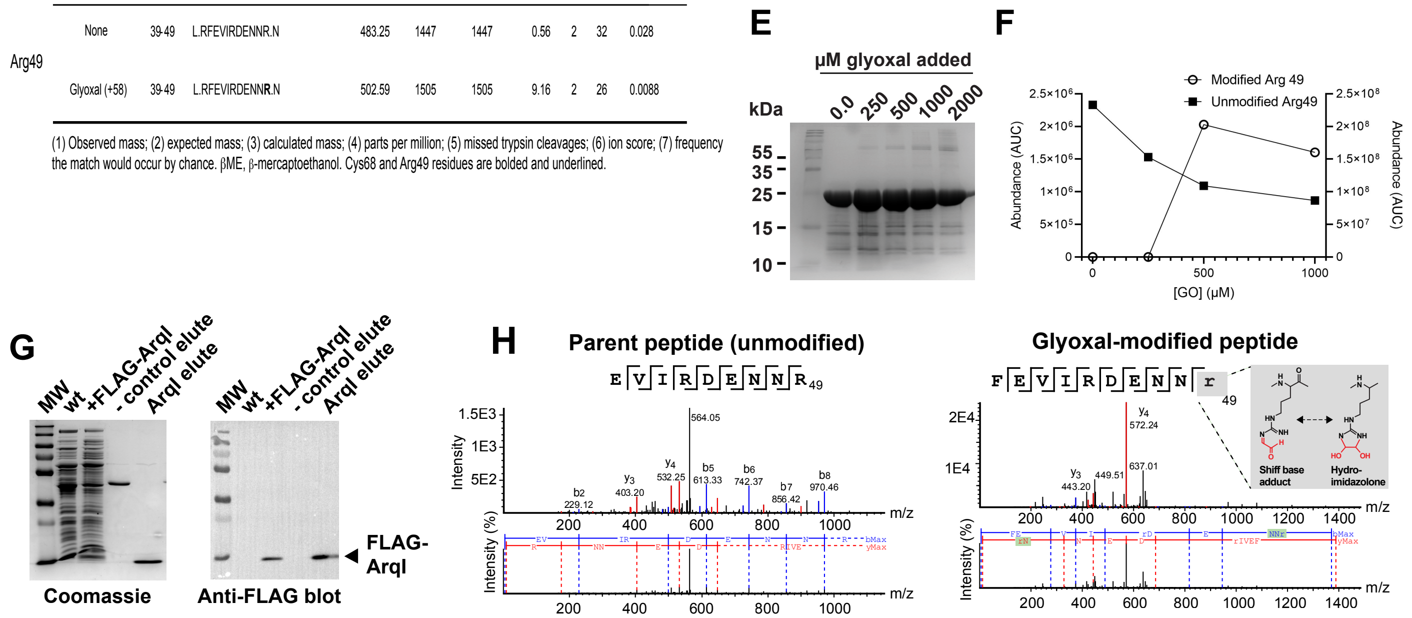

Supplementary Figure 5. **ArqI posttranslational modifications.** (**A**) Electron density map shows that Cys68 is modified by βME across each subunit (subunits A-F). The refined occupancy for Cys68 is provided. Maps are depicted as follows: 2Fo-Fc, blue, sigma 1; Fo-Fc, green and red, sigma 4. Images were generated in Coot [7] (**B**) Above: Electron density map (as described in *A*) following refinement of different glyoxal-modified Arg49 adducts modeled alongside an unmodified Arg49. Only subunits C, D, and E contained "extra Arg49" electron density. Below: Arg49 of subunits A, B, and F that did not contain extra electron density. The refined occupancies are provided for the linear adducts. (**C**) Chemical representation for the possible adducts formed when arginine reacts with GO. The reaction can result in a mass shift of +58 Daltons (Da), and can produce a hemiaminal, carboxymethylarginine, or a dihydroxyimidazolidine adduct. Alternatively, the reaction can proceed with the loss of a water molecule, resulting in a hydroimidazolone or Schiff base adduct, resulting in a +40 Da mass shift. (**D**) Table showing MS data from the ArqI Arg49/Cys68 modified crystal. (**E**) SDS-PAGE gel of purified ArqI from *E. coli* treated with increasing concentrations of GO. (**F**) Quantitation of the GO modifications using mass spectrometry (modified Arg49 versus unmodified Arg49). AUC, Area Under Curve. (**G**) Coomassie stain (left panel) and anti-FLAG western blot of immunoprecipitated (IP) FLAG-ArqI (right panel) samples used in *H* for *P. aeruginosa* mass spectrometry. (**H**) Mass spectrometry of FLAG-ArqI IP from *G*. Modified and unmodified peptides were 40 Daltons (Da) apart, indicating that a water molecule is lost in the Arg49 modified peptide in this experiment. Source data are provided as a Source Data file.

Supplementary Figure 6

A

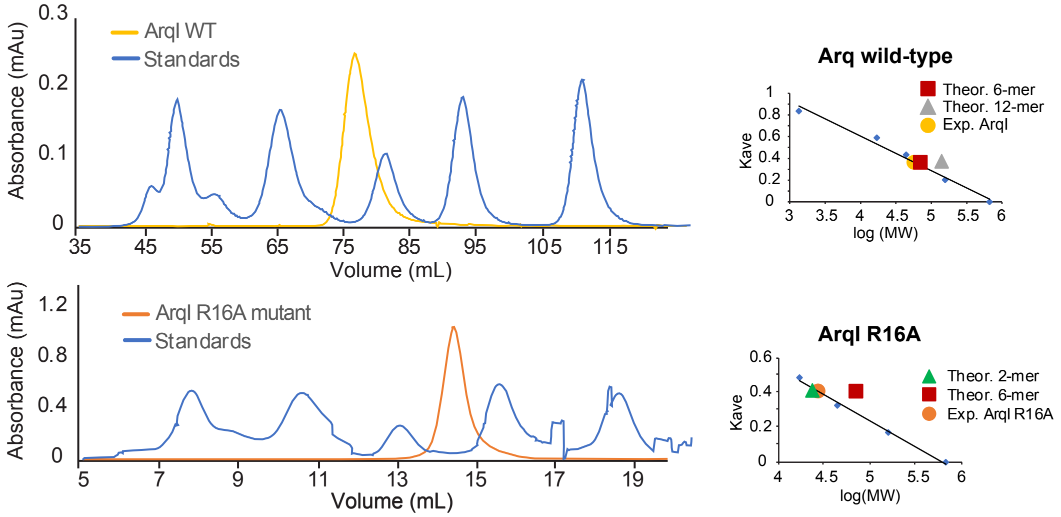

B

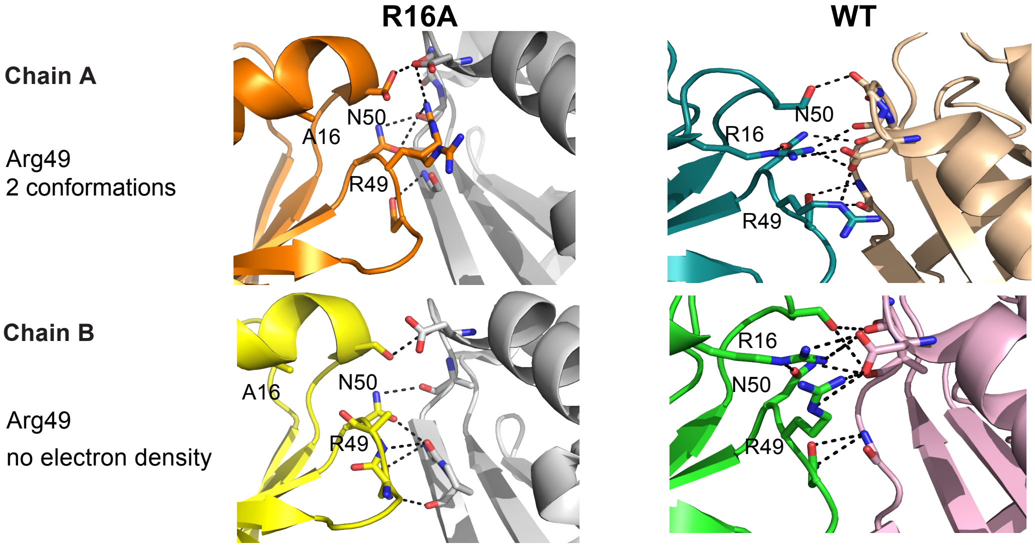

C

```
                          R16                              R49
                           ▼                                ▼
ArqI        MTYHVLVQFDVPSDKREAFAAAGLFDANGSLQNEPGTLRFEVIRDENNRNRFYLDEVYED 60
PA3390      -MYCIFIKARLRPGCAEAFIDAIQVNAAASVSTEPGCLVFDVSRDRQDPDLVYLYEVYRD 59
              * ::::  :  .  ***  *  .:* .*:..*** * *:* **.:: : .** ***.*

                  C68         D81 G86 / P87
                   ▼            ▼    ▼▼
ArqI        EAAFLQHCRNETIARFYELIDSYAFGPLFLFKGYRVEG 98
PA3390      DAAYEAHTRTAHFRDSRPLVEPLILEQEVFESDALALNPLR 100
            :**:  * *.  :     *::              *:.**
```

D

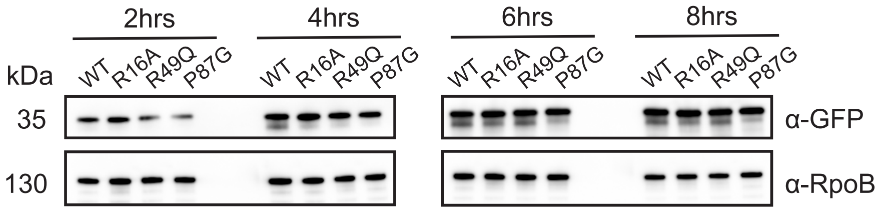

E

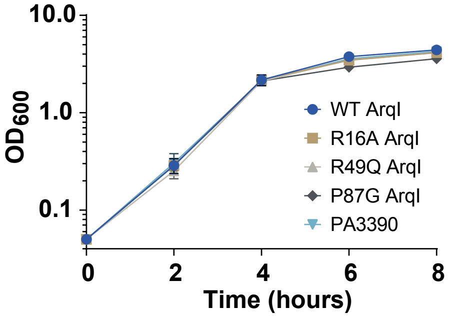

Supplementary Figure 6. **ArqI mutants and cellular localization. (A)** SEC of wild-type ArqI and R16A mutant with standards and theoretical and calculated masses. (**B**) Comparison of the R16A and WT ArqI trimeric interface from their determined atomic structures (Table S1). R16A (orange and yellow) and WT (teal and green) ArqI for chains A and B, with the neighboring asymmetric unit interacting chain shown in dark and light grey for R16A, and wheat and pink for WT. Notably, in the R16A ArqI structure, Arg49 has two conformations in chain A and is unstructured in chain B. Compared to WT ArqI, R16A has substantially fewer contacts at the trimeric interface, which might be the reason for the observed loss of the stable hexamer. (**C**) Sequence alignment of ArqI with *P. aeruginosa* control ABM domain PA3390. Notably, ArqI Arg16, Arg49, Cys68, Asp81, Gly86 and Pro87, all residues involved in interactions at the trimeric interface, are not conserved in PA3390 despite its 33% sequence identity to ArqI. Key residues of ArqI that are not present in PA3390 are highlighted and indicated by the arrows. (**D**) Western blot analysis of ArqI-sfGFP expression from the experiments in *Fig. 3*. Similar expression levels of the fusion proteins were seen across ArqI variants at the different time points. RpoB detected with a monoclonal antibody as a loading control. (**E**) Growth curve of WT and variant strains in *Fig. 3*. All strains showed similar growth kinetics, except for the ArqI-sfGFP P87G mutant, which had a slight decrease in growth at the 6- and 8-hour timepoints. n = 4 biological replicates for all groups except for P87G ArqI (n = 3 biological replicates). Whiskers denote standard deviation. Source data are provided as a Source Data file.

Supplementary Figure 7

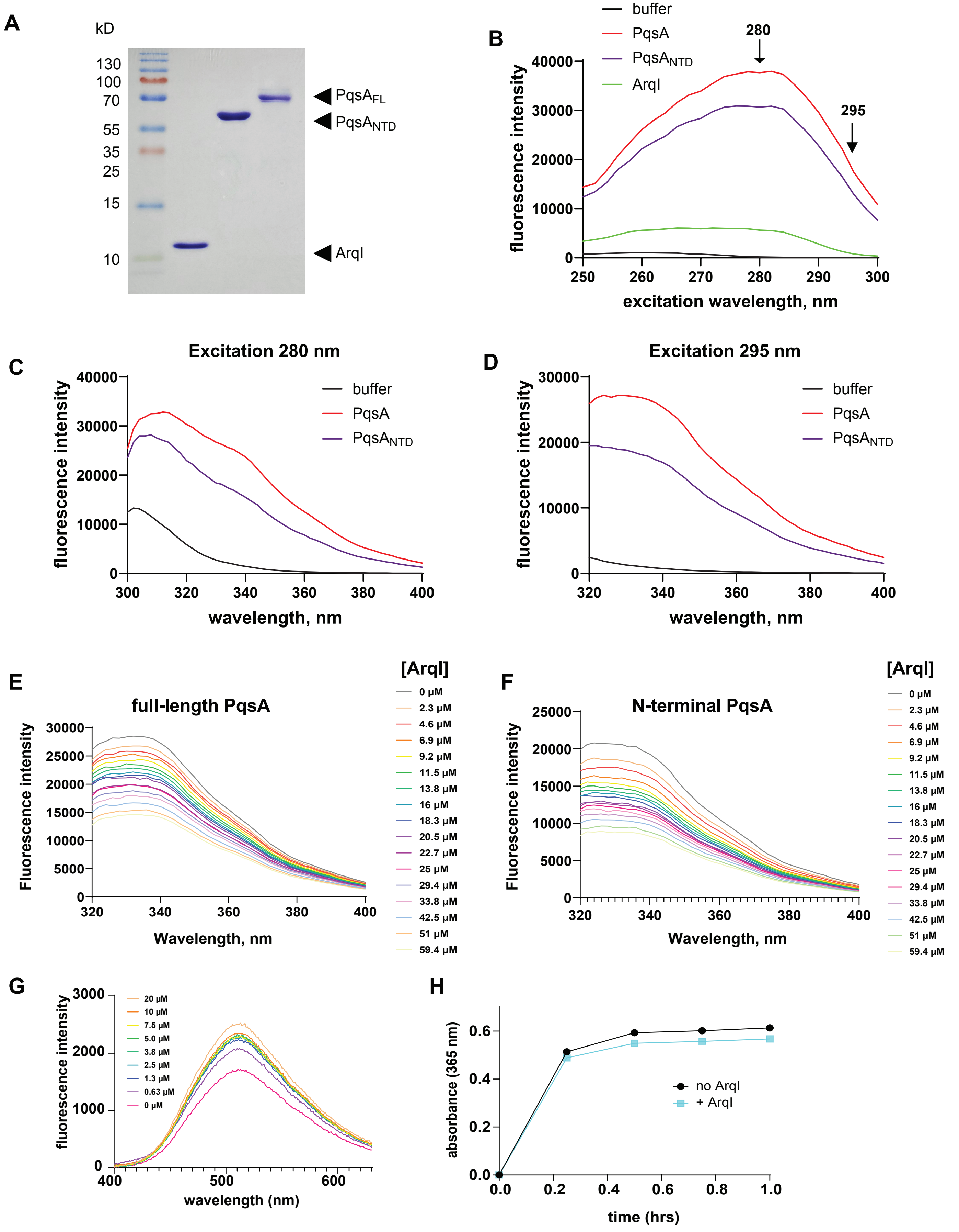

Supplementary Figure 7. Determination of PqsA-ArqI interaction by both tryptophan and Densyl quenching. (**A**) SDS-PAGE of PqsA full-length ($PqsA_{FL}$), PqsA N-terminal domain ($PqsA_{NTD}$), and ArqI wild-type purified proteins used in the quenching assays. (**B**) Excitation scan profile of quenching assay proteins (5 µM) and buffer (50 mM Tris/HCl, 150 mM NaCl, pH 8.0). Spectra of buffer control (black), ArqI (green), PqsA (red), and $PqsA_{NTD}$ (purple) each with 4.6 µM ArqI. Excitation scan was set at 250-300 nm range with emission wavelength at 332 nm. Samples were scanned in 96-well all black microtiter plates and fluorescent intensities recorded using BioTek Cytation 5 plate reader. The first arrow indicates the emission wavelength of 280 nm and showed maximum fluorescence intensities across all proteins, but also activated ArqI (green curve). At 295 nm excitation however (indicated by a second arrow), background emission from ArqI was reduced. (**C**) Emission fluorescent spectra of buffer (red), PqsA (blue), and $PqsA_{NTD}$ (black) each titrated with 4.6 µM ArqI. Excitation wavelength was set at 280 nm. (**D**) Emission fluorescent spectra of buffer (black), PqsA (red), and $PqsA_{NTD}$ (purple) each titrated with 4.6 µM ArqI. Excitation wavelength was set at 295 nm. (**E**) Determination of ArqI binding interaction to PqsA (full-length) and (**F**) $PqsA_{NTD}$ to by intrinsic tryptophan quenching. Excitation wavelength was set at 295 nm (to reduce ArqI background fluorescence as determined in *C*) and emission was scanned at a 320-400 nm range. Emission fluorescence spectra of PqsA and of $PqsA_{NTD}$ were titrated with increasing concentrations of ArqI and showed a decrease in fluorescence intensities as ArqI concentration increased. (**G**) Dansylated ArqI (DNS-ArqI) was incubated with increasing concentrations of $PqsA_{NTD}$. Results are displayed as fluorescence profiles of dansylated ArqI (5 µM) in the presence of increasing concentrations of $PqsA_{NTD}$ (0 – 20

μM). Excitation was at λ 330 nm, and emission was detected between λ 400 and 630 nm. At least three replicates were performed for all experiments. Source data are provided as a Source Data file.

Supplementary Figure 8

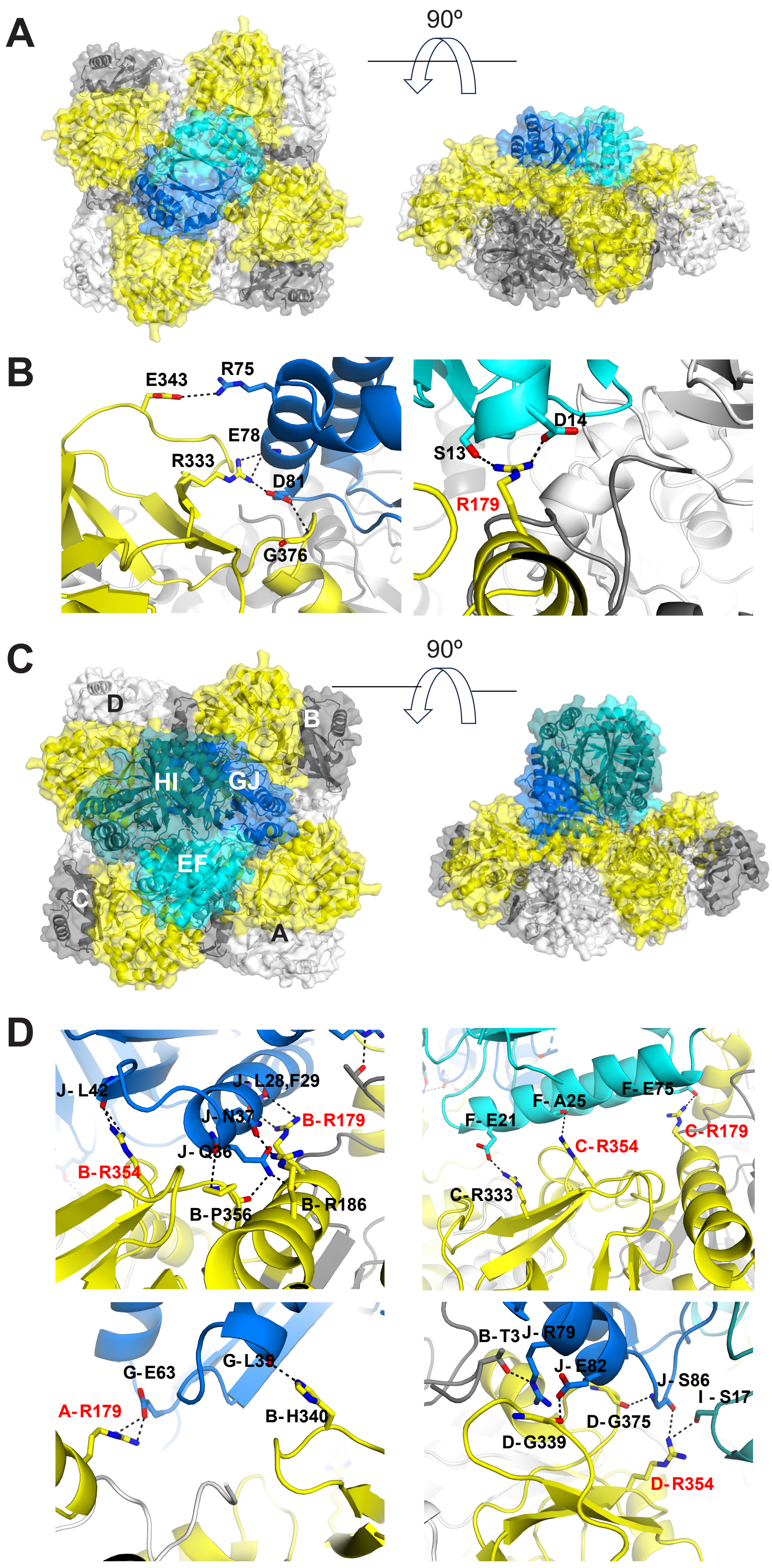

Supplementary Figure 8. Atomic modeling of the PqsA-ArqI interaction. (**A**) Top (left) and side view (right) of the PqsA tetramer bound to ArqI dimer. PqsA is colored by subunits (two are white, two are gray) with the Y2H interacting surfaces for all four subunits shown in yellow. ArqI monomers are shown in cyan (Chain F) and marine (Chain E). (**B**) H-bonds at the interface of ArqI-Chain E (top) and ArqI-Chain F (bottom). (**C**) Top view (left) and side view (right) of the ArqI hexamer interacting with PqsA. PqsA colors are as per *A*, and the three ArqI dimers are colored as follows: cyan (Chain E/F), marine (Chain G/J), and deep teal (Chain H/I). (**D**) Relevant H-bonding between the ArqI hexamer subunits and PqsA. Models were generated using either AlphaFold3 [8] or HDOCK [6]as described in the text.

Supplementary Figure 9

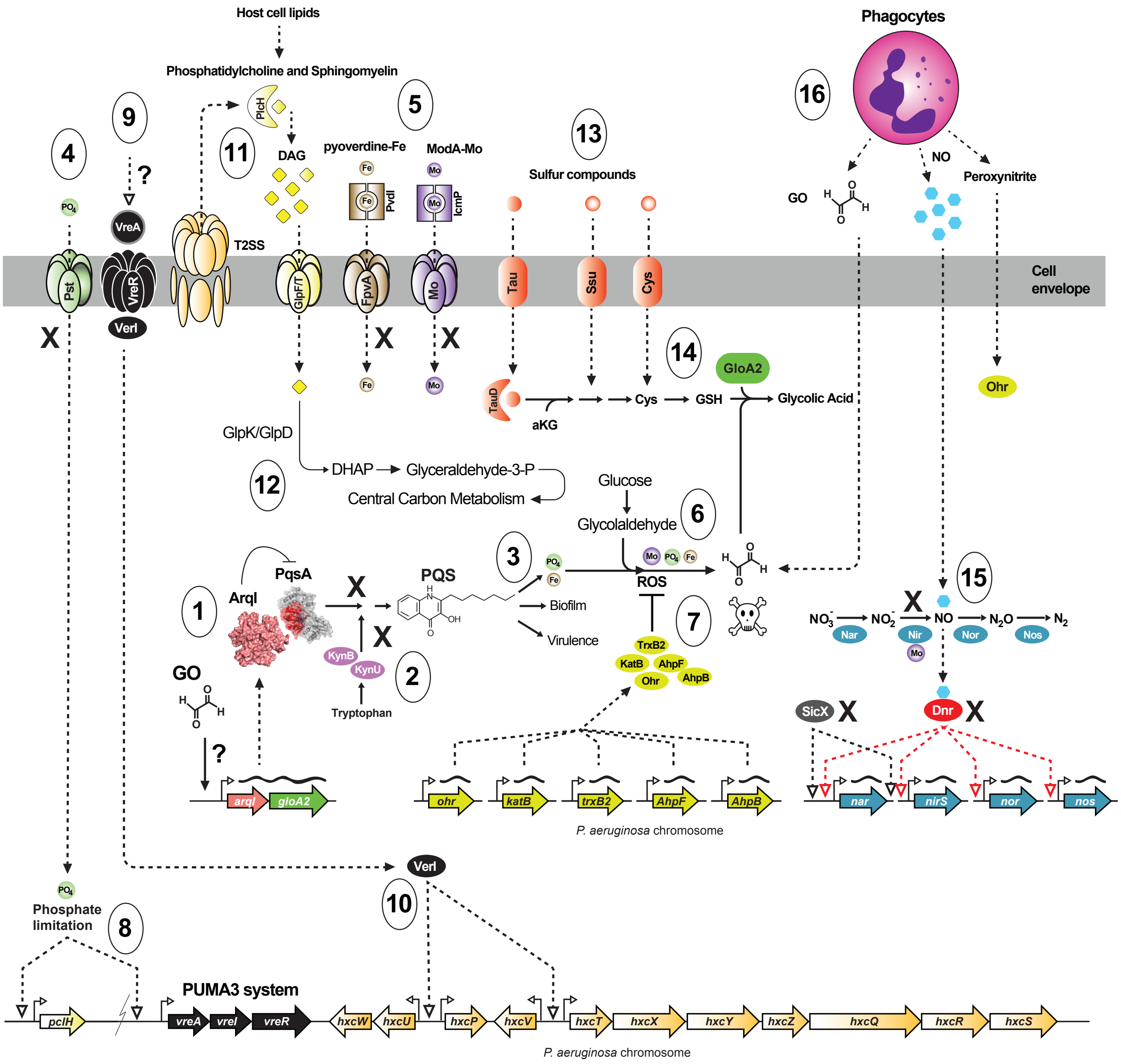

Supplementary Figure 9. Overview of the ArqI-GloA2 and GO response in *P. aeruginosa*. (**1**) When glyoxal is present in the cell, the *arqI-gloA2* operon is transcribed and translated. ArqI production results in its biding to PqsA and a halt in PQS biosynthesis by an as-of-yet unknown mechanism. (**2**) In addition, the second PQS biosynthesis metabolic route is repressed by the downregulation of enzymes KynB and KynU. (**3**) PQS-associated iron uptake and its specific virulence traits [9] would therefore be halted. (**4**) Phosphate (P), (**5**) iron (Fe) and molybdenum (Mo) uptake are repressed which would otherwise react with glycolaldehyde to form more GO, and (**7**) ROS remediation enzymes (Ohr, KatB, TrxB2, AhpF/B) are upregulated to further prevent ROS generated GO. The downregulation of the Pst phosphate uptake and sensory system results in phosphate starvation, (**8**) synthesis of the lipase PlcH and activation of *vreAIR* (*PUMA3*) extracellular sigma factor operon (**9**) whose inducing signal is currently unknown. (**10**) VreI becomes active and initiates the *hxc* T2SS operon, and (**11**) release of the lipase PlcH. PlcH cleaves host phosphatidyl choline to yield DAG (**12**) which could be taken up and processed by the Glp operon to eventually enter the TCA cycle. (**13**) Host taurine and other sulfur-bearing compounds are internalized to help replenish the sulfur pool (**14**), and would eventually provide the GSH substrate for GloA2 to detoxify GO into the more harmless glycolic acid carbon source, which recycles into the TCA cycle. (**15**) Also strongly downregulated is the Mo-utilizing enzyme nitrite reductase NirS, the small RNA *sicX* and the dissimilative nitrate respiration regulator (Dnr) - all of which control the denitrification pathway. Conversely, the Nor/Nos pathway is upregulated to allow detoxification of nitric oxide (NO), (**16**) which is a well-established means by which phagocytes kill bacteria [10-12]. Several publications have implicated the aldehyde

remediation response as critical for neutrophil effectiveness during sepsis. GO, glyoxal; αKG, alpha ketoglutarate; T2SS, Type II Secretion System; NO, nitric oxide; DAG, diacylglycerol; X, pathway is repressed by GO addition.

Supplementary Figure 10

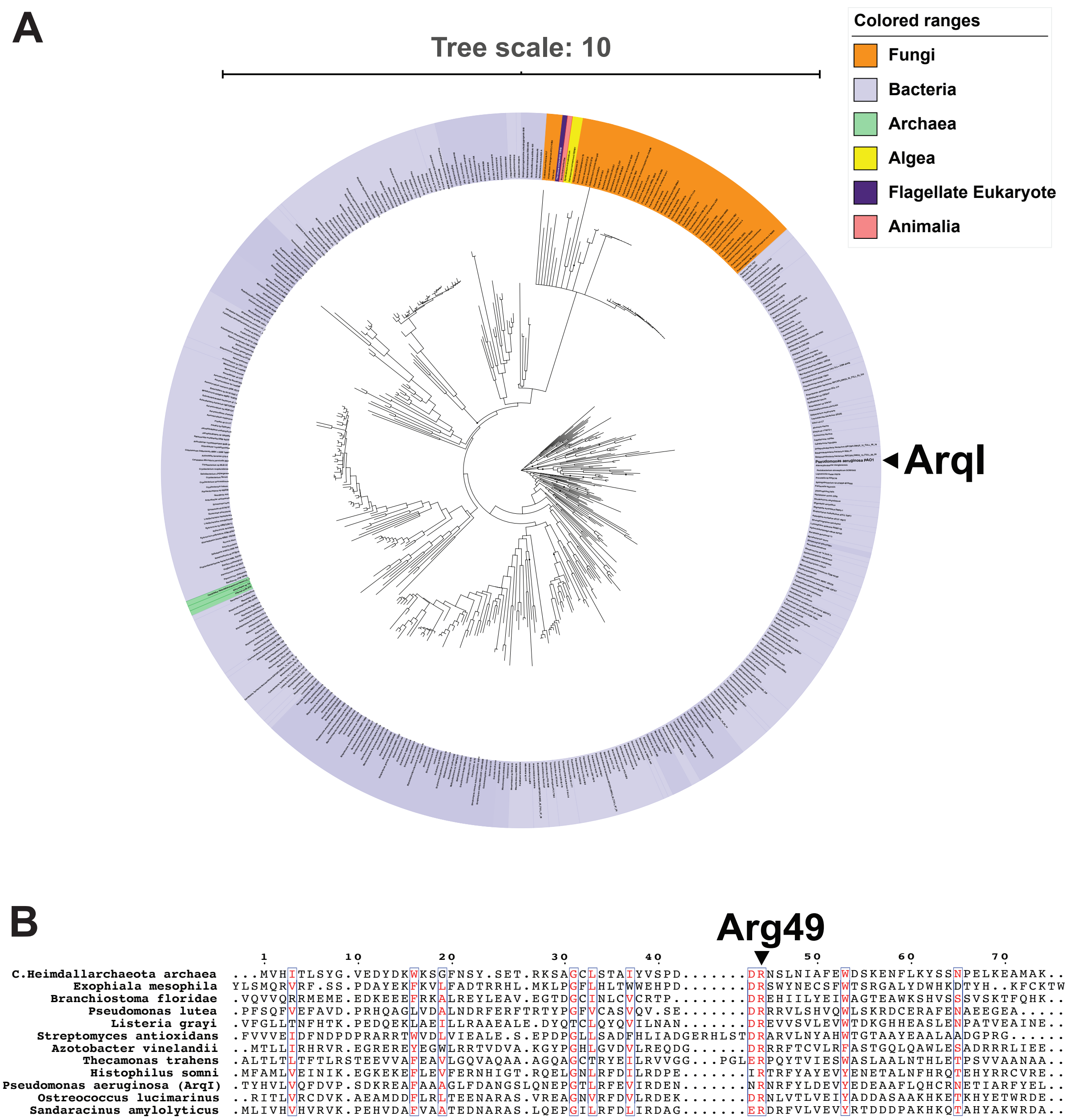

Supplementary Figure 10. **Conservation of Arg49 in diverse species**. (**A**) ABM domains containing Arg49 were searched from the UniProt database (PF03992) and aligned using the HMMER Biosequence online analysis tool [13] and displayed with iTOL [14]. Results produced 441 ABM domain containing sequences from diverse phyla / species, including a few from unicellular eukaryotes (*e.g.* the photosynthetic microalgae *Symbiodinium microadriaticum, and eukaryotic Flagellate Thecamonas trahens*) and one sequence from a lancet, the fish-like chordate *Branchiostoma floridae*. There were also several fungi and Archaea sequences found that contain an Arg49 equivalent residue. All sequences are given in **Table 2, SI**. (**B**) Alignment of a subset of environmental sequences from *A* and ArqI (*P. aeruginosa*). The conserved Arg49 is noted with an arrow.

Supplementary References

1 Szklarczyk, D. *et al.* The STRING database in 2023: protein-protein association networks and functional enrichment analyses for any sequenced genome of interest. *Nucleic Acids Res* **51**, D638-D646 (2023). https://doi.org:10.1093/nar/gkac1000

2 Muller, I. *et al.* Crystal structure of the alkylsulfatase AtsK: insights into the catalytic mechanism of the Fe(II) alpha-ketoglutarate-dependent dioxygenase superfamily. *Biochemistry* **43**, 3075-3088 (2004). https://doi.org:10.1021/bi035752v

3 Sievers, F. *et al.* Fast, scalable generation of high-quality protein multiple sequence alignments using Clustal Omega. *Mol Syst Biol* **7**, 539 (2011). https://doi.org:10.1038/msb.2011.75

4 Robert, X. & Gouet, P. Deciphering key features in protein structures with the new ENDscript server. *Nucleic Acids Res* **42**, W320-324 (2014). https://doi.org:10.1093/nar/gku316

5 Robinson, J. T. *et al.* Integrative genomics viewer. *Nat Biotechnol* **29**, 24-26 (2011). https://doi.org:10.1038/nbt.1754

6 Yan, Y., Zhang, D., Zhou, P., Li, B. & Huang, S. Y. HDOCK: a web server for protein-protein and protein-DNA/RNA docking based on a hybrid strategy. *Nucleic Acids Res* **45**, W365-W373 (2017). https://doi.org:10.1093/nar/gkx407
7 Emsley, P. & Cowtan, K. Coot: model-building tools for molecular graphics. *Acta Crystallogr D Biol Crystallogr* **60**, 2126-2132 (2004). https://doi.org:10.1107/S0907444904019158
8 Jumper, J. *et al.* Highly accurate protein structure prediction with AlphaFold. *Nature* **596**, 583-589 (2021). https://doi.org:10.1038/s41586-021-03819-2
9 Lin, J. *et al.* A Pseudomonas T6SS effector recruits PQS-containing outer membrane vesicles for iron acquisition. *Nat Commun* **8**, 14888 (2017). https://doi.org:10.1038/ncomms14888
10 Akbari, M. S., Joyce, L. R., Spencer, B. L., McIver, K. S. & Doran, K. S. Identification of Glyoxalase A in Group B Streptococcus and its contribution to methylglyoxal tolerance and virulence. *bioRxiv* (2024). https://doi.org:10.1101/2024.07.30.605887
11 Zhang, M. M., Ong, C. L., Walker, M. J. & McEwan, A. G. Defence against methylglyoxal in Group A Streptococcus: a role for Glyoxylase I in bacterial virulence and survival in neutrophils? *Pathog Dis* **74** (2016). https://doi.org:10.1093/femspd/ftv122
12 Liu, X. *et al.* Glyoxal oxidase-mediated detoxification of reactive carbonyl species contributes to virulence, stress tolerance, and development in a pathogenic fungus. *PLoS Pathog* **20**, e1012431 (2024). https://doi.org:10.1371/journal.ppat.1012431
13 Finn, R. D., Clements, J. & Eddy, S. R. HMMER web server: interactive sequence similarity searching. *Nucleic Acids Res* **39**, W29-37 (2011). https://doi.org:10.1093/nar/gkr367
14 Letunic, I. & Bork, P. Interactive Tree Of Life (iTOL): an online tool for phylogenetic tree display and annotation. *Bioinformatics* **23**, 127-128 (2007). https://doi.org:10.1093/bioinformatics/btl529
