# Supplementary material for: A glyoxal-specific aldehyde signaling axis in Pseudomonas aeruginosa that influences quorum sensing and infection
Source: Nat Commun. 2025 Jul 18;16:6616. doi: 10.1038/s41467-025-61469-8 (PMC12274486; doi:10.1038/s41467-025-61469-8)
Supplement: Supplementary file 2 — Description of Additional Supplementary File [file 41467_2025_61469_MOESM2_ESM.pdf]

Description of Additional Supplementary Files

Supplementary Data 1. STRING and RNA-seq data. For STRING data Not Treated (NT) versus glyoxal (GO) treated outputs are given with 3- and 5-fold cutoffs (average log2 change) for the original combined output file, and separately, files with up-regulated and down-regulated genes with GO treatment (15 minute and 1-hour timepoints combined; S1C-S1D). For RNA-seq data files are given for GO 15 minute and 1-hour treatments (S1B-S1A), and a separate file comparing 15-minute versus 1-hour overlapping genes that were differentially expressed (S1E). Non-overlapping genes are highlighted in orange.

Supplementary Data 2. Fasta file of ABM domains containing an ArqI Arg49 residue.

Supplementary Data 3. Table of strains, plasmids and oligos.

Supplementary Data 4. Fasta file of ABM domains predicted to be translationally fused to glyoxalase domains.

Supplementary Data 5. Fasta file of ABM binding domains predicted to be translationally fused to AMP binding domains.
