# Supplementary material for: A glyoxal-specific aldehyde signaling axis in Pseudomonas aeruginosa that influences quorum sensing and infection
Source: Nat Commun. 2025 Jul 18;16:6616. doi: 10.1038/s41467-025-61469-8 (PMC12274486; doi:10.1038/s41467-025-61469-8)
Supplement: Supplementary file 5 — Supplementary Dataset 3 [file 41467_2025_61469_MOESM5_ESM.docx]

**Table 3. Strains, plasmids and oligos used in this study.**

| Strain | Description | Reference |
| --- | --- | --- |
| *E. coli* | | |
| DH5α | Standard E. coli cloning strain. | Lab stock |
| CC118 λpir | E. coli strain for maintenance of pKNG101 derived plasmids. | A. Filloux lab stock |
| T7 Express *lysY^i/q^* | E. coli strain for recombinant protein expression. | New England Biolabs, Ipswich, MA |
| LOBSTR | E. coli strain for recombinant protein expression. | Lab Stock |
| SM10 λpir | P. aeruginosa conjugation donor strain A. Filloux lab stock Expresses the λpir protein, allowing replication of suicide (Simon et al., 1983) plasmids with the R6K origin of replication. Kanamycin resistant. | A. Filloux lab stock (Simon et al., 1983) |
| SM10 λpir pTNS3 | P. aeruginosa conjugation transposase donor strain. | Lab stock |
| 1047 pRK2013 | Conjugation helper strain. Kanamycin resistant. | Lab stock |
| *P. aeruginosa* | | |
| MPAO1 | WT lab strain | Manoil Lab. (Holloway, 1955; Jacobs et al., 2003; Stover et al., 2000) |
| MPAO1 Δ*arqI-gloA2* | Clean deletion of the *arqI-gloA2* operon | This work |
| MPAO1 Δ*arqI-gloA2* Tn7::P_arqI_-*arqI*-FLAG | FLAG-tagged *arqI* complement under the control of the *arqI* native promoter at the Tn7 site | This work |
| MPAO1 Δ*arqI-gloA2* Tn7::P*_arqI_*-*gloA2*-HA | HA-tagged *gloA2* complement under the control of the *arqI* native promoter at the Tn7 site | This work |
| MPAO1 Δ*arqI-gloA2* Tn7::P*_arqI_*-*arqI*-FLAG-*gloA2*-HA | FLAG-tagged *arqI* and HA-tagged *gloA2* complement under the control of the *arqI* native promoter at the Tn7 site | This work |
| MPAO1 *attB*::PA1/04/03-mCherry | MPAO1 constitutively expressing mCherry | This work |
| MPAO1 *attB*::PA1/04/03-sfGFP | MPAO1 constitutively expressing sfGFP | This work |
| MPAO1 *attB*::P*pqsA*-mCherry | MPAO1 with mCherry fused to the *pqsA* promoter at the *attB* site. PQS biosensor. | This work |
| Plasmid | **Description** | **Reference** |
| pSB109 | Low copy plasmid harboring an arabinose-inducible promoter. Gentamicin resistant. | Bardy Lab, Ketelboeter et al. 2017 |
| pSB109-FLAG-ArqI | Arabinose-inducible plasmid for FLAG-ArqI expression. | This work |
| pSB109-ArqI | Arabinose-inducible plasmid for untagged ArqI expression. | This work |
| pSB109-ArqI-sfGFP | Arabinose-inducible plasmid for arqI-sfGFP localization. | This work |
| pSB109-ArqI-sfGFP R16A | R16A mutation of pSB109-ArqI-sfGFP | This work |
| pSB109-ArqI-sfGFP R49Q | R49Q mutation of pSB109-ArqI-sfGFP | This work |
| pSB109-ArqI-sfGFP P87G | P87G mutation of pSB109-ArqI-sfGFP | This work |
| pSB109-PA3390-sfGFP | Arabinose-inducible plasmid for PA3390-sfGFP localization. | This work |
| pSB109-GloA2-HA | Arabinose-inducible plasmid for HA-tagged GloA2 expression. | This work |
| pSB109-ArqI-FLAG-GloA2-HA | Arabinose-inducible plasmid for ArqI and GloA2 expression. | This work |
| pSB109-PA3390-FLAG | Arabinose-inducible expression of FLAG-tagged PA3390 | This work |
| pCC21 | Low copy empty plasmid derived from pSB109. | This work |
| pCC21-P*arqI* | *arqI* Promoter in pCC21 | This work |
| pCC21-P*arqI*-mScarlet-I | Fluorescent reporter construct for ArqI expression. | This work |
| pCC21-P*arqI*-ArqI-mScarlet-I | mScarlet-I tagged *arqI* under the control of its native promoter. | This work |
| pCC21-P*arqI*-sfGFP | mScarlet-I tagged ArqI under the control of its native promoter. | This work |
| mini-CTX1-Gm | Plasmid for insertion at *attB* site | Allen Lab, Hoang et al. 2000 |
| mini-CTX1-Gm-P*pqsA*-mCherry | mCherry under control of *pqsA* promoter. PQS biosensor. | This work |
| mini-CTX1-Gm-PA1/04/03-mCherry | Plasmid to fluorescently mark strains with mCherry | This work |
| mini-CTX1-Gm-PA1/04/03-sfGFP | Plasmid to fluorescently mark strains with sfGFP | This work |
| pMMB67EH | IPTG-inducible, low copy plasmid. Ampicillin resistant. | Allen Lab, Furste et al. 1986 |
| pMMB67EH-FLAG | pMMB67EH with enhanced RBS and integrated FLAG tag. | This work |
| pMMB67EH-PqsA-HA | IPTG-inducible expression of HA-tagged PqsA | This work |
| pSB109-ArqI-BirA*-FLAG | ArqI-BirA* fusion expression plasmid. Arabinose inducible. | This work |
| pSB109-BirA*-FLAG | BirA* expression plasmid. Arabinose inducible. | This work |
| pE-SUMO | Protein expression plasmid with N-terminal SUMO tag. | Life Sensors Inc. |
| pE-SUMO-ArqI | ArqI expression plasmid with N-terminal SUMO tag. | This work |
| pE-SUMO-ArqI R16A | R16A mutation of pE-SUMO-ArqI | This work |
| pE-SUMO-ArqI R49Q | R49Q mutation of pE-SUMO-ArqI | This work |
| pE-SUMO-ArqI P87G | P87G mutation of pE-SUMO-ArqI | This work |
| pKNG101 | Suicide vector for gene deletions. Streptomycin resistant. | Muhl and Filloux, 2014 |
| pKNG101-*arqI-gloA2* | Suicide vector for deletion of the *arqI-gloA2* operon | This work |
| pUC18T-mini-Tn7T-Gm | Tn7 insertion plasmid. | Choi and Schweizer, 2006 |
| pUC18T-mini-Tn7T-Gm-*arqI*-FLAG | FLAG-tagged ArqI complement under the control of the arqI native promoter at the Tn7 site | This work |
| pUC18T-mini-Tn7T-Gm-*gloA2*-HA | HA-tagged GloA2 complement under the control of the arqI native promoter at the Tn7 site | This work |
| pUC18T-mini-Tn7T-Gm-*arqI*-FLAG-*gloA2*-HA | FLAG-tagged arqI and HA-tagged gloA2 complement under the control of the *arqI* native promoter at the Tn7 site | This work |
| pCPD | Protein expression vector with cleavable CPD tag | Biancucci et al. 2017 |
| pCPD-PqsA | PqsA expression plasmid with N-terminal CPD tag | This work |
| pQLinkG2 | Expression plasmid with N-terminal GST tag | Scheich et al. 2007 |
| pQLinkG2-PqsA-NTD | PqsA N-terminal Domain expression plasmid with N-terminal GST tag | This work |
| pFLP2 | Vector expressing flippase for removal of antibiotic resistance cassettes from *attb* and Tn7 integrated constructs | Hoang et al. 1998 |
| Description | **Oligo Name** | **Nucleotide Sequence (5'->3')** |
| To construct pSB109-FLAG-ArqI | NcoI-FLAG-*arqI*-F1 | CATGGATGGATTATAAAGATGATGACGACAAAATGACCTACCACGTACTGGTTCA |
|  | NcoI-FLAG-*arqI*-F2 | GATGGATTATAAAGATGATGACGACAAAATGACCTACCACGTACTGGTTCA |
|  | NdeI-*arqI*-R1 | TATGTCAACCCTCGACGCGGTAG |
|  | NdeI-*arqI*-R2 | TGTCAACCCTCGACGCGGTAG |
| To construct pSB109-ArqI | PstI-*arqI*-No-Tag-R1 | TGCAGTCAACCCTCGACGCGGTAG |
|  | PstI-*arqI*-No-Tag-R2 | GTCAACCCTCGACGCGGTAG |
| To construct pSB109-PA3390-FLAG | NcoI-*PA3390*-F1 | CATGGATGTACTGCATCTTTATCAAGGCCC |
|  | NcoI-*PA3390*-F2 | GATGTACTGCATCTTTATCAAGGCCC |
|  | NdeI-*PA3390*-FLAG-R1 | TATGTCATTTGTCGTCATCATCTTTATAATCGCGCAGCGGATTGAGCGC |
|  | NdeI-*PA3390*-FLAG-R2 | TGTCATTTGTCGTCATCATCTTTATAATCGCGCAGCGGATTGAGCGC |
| To construct pSB109-ArqI-sfGFP | NdeI-sfGFP-R1 | TATGTTACTTATAAAGCTCATCCATGCCG |
|  | NdeI-sfGFP-R2 | TGTTACTTATAAAGCTCATCCATGCCG |
| To construct pCC21-P*_arqI_*-*arqI*-sfGFP | BamHI-145bp-UP-*arqI*-F | GGTGGTGGATCCGCAGAAGAATCCGAGTTGAGAG |
|  | Linker-*arqI*-R | GCTAGTCCCTGCTTTTGCAGCAGCCTCACCACCAGATCCGCGACCCTCGACGCGGTAGCC |
|  | sfGFP-Linker-F | GCTGCAAAAGCAGGGACTAGCATGTCAAAAGGAGAAGAGCTGTTCAC |
|  | HindIII-sfGFP-R | GGTGGTAAGCTTTTACTTATAAAGCTCATCCATGCCGTG |
| To construct pSB109-PA3390-sfGFP | sfGFP-Linker-F | GCTGCAAAAGCAGGGACTAGCATGTCAAAAGGAGAAGAGCTGTTCAC |
|  | Linker-*PA3390*-R | GCTAGTCCCTGCTTTTGCAGCAGCCTCACCACCAGATCCGCGGCGCAGCGGATTGAGCGC |
| To construct pSB109-GloA2-HA | NcoI-*gloA2*-F1 | CATGCGAATCCTGCACAGCATGC |
|  | NcoI-*gloA2*-F2 | CGAATCCTGCACAGCATGCTC |
|  | PstI-*gloA2*-R1 | TGCAGTCAAGCATAATCGGGCACGTCG |
|  | PstI-*gloA2*-R2 | GTCAAGCATAATCGGGCACGTCG |
| To construct pUC18T-mini-Tn7T-Gm-*arqI*-FLAG-*gloA2*-HA | *arqI*-FLAG-Link-R | GATCATTTGTCGTCATCATCTTTATAATCACCCTCGACGCGGTAGC |
|  | *arqI*-FLAG-Link-F | GATTATAAAGATGATGACGACAAATGATCCCCGCTCCGGCG |
|  | HindIII-*gloA2*-HA-R | GGTAAGCTTTCAAGCATAATCGGGCACGTCGTAGGGGTAATCGAACTGGGTGCCCTTCTG |
| To construct pUC18T-mini-Tn7T-Gm-*gloA2*-HA | *arqI*-145-up-LINK-*gloA2*-R | GATTCGCATGGAAACTCTCCGTCGTGAAATGGGGTTGGAAGC |
|  | *arqI*-145-up-LINK-*gloA2*-F | GCTTCCAACCCCATTTCACGACGGAGAGTTTCCATGCGAATC |
| To construct pUC18T-mini-Tn7T-Gm-*arqI*-FLAG | HindIII-*arqI*-FLAG-R | GGTGGTaagcttTCATTTGTCGTCATCATCTTTATAATCACCCTCGACGCGGTAGC |
| To construct pCC21 | pSB109-seq-F-MCS (synthesized dsDNA fragment) | GAAAAGTCCACATTGATTATTTGCACGGCGTCACACTTTGCTATGCCATAGCATTTTTATCCATAAGATTAATTAACCCTCACTAAAGGGAACAAAAGCTGGAGCTCCACCGCGGTGGCGGCCGCTCTAGAACTAGTGGATCCCCCGGGCTGCAGGAATTCGATATCAAGCTTATCGATACCGTCGACCTCGAGGGGGGGCCCGGTACC |
| To construct pCC21-P*_arqI_*-mScarlet-I | SacI-P*arqI*-F | CCGCCGGAGCTCGCAGAAGAATCCGAGTTGAGAG |
|  | XbaI-P*arqI*-R | CGGCGGTCTAGACCATGGAATCTTTCCTCGTGAAATG |
|  | NcoI-mScarlet-I-F1 | CATGGTTAGCAAAGGTGAGGCAG |
|  | NcoI-mScarlet-I-F2 | GTTAGCAAAGGTGAGGCAGTG |
|  | KpnI-mScarlet-I-R1 | GTACCTCATTTATAAAGCTCGTCCATGCCCC |
|  | KpnI-mScarlet-I-R2 | CTCATTTATAAAGCTCGTCCATGCCCC |
| To construct pCC21-P*_arqI_*-*arqI*-mScarlet-I | mScarlet-I-Linker-F | GCTGCAAAAGCAGGGACTAGCATGGTTAGCAAAGGTGAGGCAG |
|  | HindIII-mScarlet-I-R. | GGTGGTAAGCTTTCATTTATAAAGCTCGTCCATGCCCC |
| To construct mini-CTX1-Gm-PA1/04/03-mCherry | HindIII-RBS-mCherry-KpnI (synthesized dsDNA fragment) | GATATCAAGCTTTAGCGAAGGAGATATACCATGGTCTCGAAGGGGGAAGAAGATAACATGGCCATCATCAAGGAATTCATGCGCTTCAAGGTCCACATGGAGGGGAGCGTGAATGGCCACGAATTCGAAATCGAAGGGGAAGGCGAAGGCCGCCCCTACGAAGGCACCCAGACCGCCAAACTCAAGGTGACGAAAGGCGGGCCGCTGCCCTTCGCCTGGGACATCCTCAGCCCGCAATTCATGTATGGGTCCAAGGCCTACGTCAAGCACCCGGCGGACATCCCGGACTACCTCAAACTGTCCTTCCCCGAAGGCTTCAAGTGGGAGCGCGTGATGAATTTCGAAGACGGGGGCGTCGTGACCGTCACGCAGGATAGCTCCCTCCAAGACGGCGAGTTCATCTACAAGGTCAAACTCCGGGGGACGAACTTCCCCTCGGATGGCCCCGTGATGCAAAAAAAGACGATGGGGTGGGAGGCCTCGTCGGAACGCATGTATCCCGAAGATGGCGCCCTGAAAGGCGAGATCAAACAGCGCCTGAAGCTCAAAGACGGGGGGCATTATGATGCCGAAGTGAAAACCACCTACAAGGCCAAGAAGCCCGTGCAGCTGCCGGGCGCCTATAATGTGAACATCAAGCTCGATATCACGAGCCACAACGAGGATTATACGATCGTGGAGCAGTACGAGCGGGCGGAGGGCCGGCATAGCACGGGCGGGATGGACGAGCTGTATAAATGAGGTACCCAATTC |
|  | BamHI-PA1/04/03-NcoI | GATCCAAAATTTATCAAAAAGAGTGTTGACTTGTGAGCGGATAACAATGATACTTAGATTCAATTGTGAGCGGATAACAATTTCACACATCTAGAATTAAAGAGGAGAAATTAAG |
|  | BamHI-PA1/04/03-NcoI-RC | CATGCTTAATTTCTCCTCTTTAATTCTAGATGTGTGAAATTGTTATCCGCTCACAATTGAATCTAAGTATCATTGTTATCCGCTCACAAGTCAACACTCTTTTTGATAAATTTTG |
| To construct mini-CTX1-Gm-PA1/04/03-sfGFP | NcoI-sfGFP-F1 | CATGGTAAAAGGAGAAGAGCTGTTCAC |
|  | NcoI-sfGFP-F2 | GTAAAAGGAGAAGAGCTGTTCAC |
|  | sfGFP-KpnI-R1 | GTACCTTACTTATAAAGCTCATCCATGCCGTG |
|  | sfGFP-KpnI-R2 | CTTACTTATAAAGCTCATCCATGCCGTG |
| To construct pMMB67EH-FLAG | pSB109-MCS-FLAG (synthesized dsDNA fragment) | GAAACAGAGCTCGAAGGAGATATAGCATGCGCAGCAGCCAGGATCCGATTATAAAGATGATGACGACAAATGACGCGCGGCAGCCATATGGAATTCCTGCAGTACCCGTACGACGTGCCGGACTACGCCTGACCCGGGAAGCTTTAAACT |
| To construct pMMB67EH-PqsA-HA | SphI-*pqsA*-F1 | CATGTCCACATTGGCCAACCTG |
|  | SphI-*pqsA*-F2 | TCCACATTGGCCAACCTGACC |
|  | *pqsA*-PstI-R1 | TGCAGACATGCCCGTTCCTCCGG |
|  | *pqsA*-PstI-R2 | GACATGCCCGTTCCTCCGG |
| To construct pE-SUMO-ArqI | BsaI-*arqI*-F1 | AGGTAGCGGAGGTGGAATGACCTACCACGTACTGGTTC |
|  | BsaI-*arqI*-F1 | AGCGGAGGTGGAATGACCTACCACGTACTGGTTC |
|  | BsaI-*arqI*-R1 | CTAGTCAACCCTCGACGCGGTAG |
|  | BsaI-*arqI*-R2 | TCAACCCTCGACGCGGTAG |
| To construct pKNG101-*arqI-gloA2* | *arqI*-500bp-Up-F | GATCCAGCAGCAGCGAATGCTCCG |
|  | BamHI-*arqI-gloA2*-F2 | CAGCAGCAGCGAATGCTCCG |
|  | SpeI-*arqI-gloA2*-R1 | CTAGTATCGACGCCATCGCCCAC |
|  | SpeI-*arqI-gloA2*-R2 | TATCGACGCCATCGCCCAC |
|  | *arqI-gloA2*-check-F | TGGCCAGCAACGGGTGTTC |
|  | *arqI-gloA2*-check-R | GTTCCGAGGAGTGCTTGCATG |
| Site Directed Mutagenesis | *arqI*-R16A-F | GTTCCCTCCGACAAGGCCGAAGCGTTCGCCGCGG |
|  | *arqI*-R16A-R | CCGCGGCGAACGCTTCGGCCTTGTCGGAGGGAAC |
|  | *arqI*-R49Q-F | CGCGACGAGAACAACCAAAACCGCTTCTACCTC |
|  | *arqI*-R49Q-R | GAGGTAGAAGCGGTTTTGGTTGTTCTCGTCGCG |
|  | *arqI*-P87G-F | TTCGGTGGGCTTTTCCTGTTCAAG |
|  | *arqI*-P87G-F | GAAAAGCCCACCGAAGGCGTAGC |
| Synthesized mScarlet-I dsDNA Fragment | mScarlet-I | ATGGTTAGCAAAGGTGAGGCAGTGATAAAAGAGTTCATGCGCTTCAAGGTTCACATGGAGGGCTCTATGAATGGTCATGAGTTTGAAATCGAAGGGGAAGGGGAAGGCCGTCCTTACGAAGGGACACAGACCGCGAAGTTAAAAGTCACGAAAGGCGGGCCATTACCATTCAGTTGGGACATATTGAGTCCACAATTTATGTACGGAAGCCGAGCGTTTATTAAACACCCTGCCGACATCCCAGATTACTACAAACAATCTTTCCCAGAAGGCTTCAAATGGGAACGCGTCATGAATTTCGAAGATGGTGGGGCGGTCACGGTAACCCAGGACACATCACTTGAGGACGGTACGTTGATATACAAGGTCAAACTACGAGGGACTAACTTCCCGCCGGATGGCCCAGTCATGCAAAAGAAGACTATGGGATGGGAAGCGTCTACCGAGCGATTATACCCTGAGGACGGAGTGCTAAAGGGCGACATCAAGATGGCGTTGCGACTTAAAGATGGTGGTAGATATTTAGCAGATTTTAAAACAACGTATAAAGCTAAAAAACCAGTCCAAATGCCGGGAGCATACAACGTGGACAGAAAACTAGACATCACATCACACAATGAGGATTATACTGTGGTCGAGCAGTATGAGCGTTCAGAAGGCCGTCATAGTACTGGGGGCATGGACGAGCTTTATAAATGA |
| To construct mini-CTX1-Gm-P*_pqsA_*-mCherry | BamHI-*pqsA*-Prom-F1 | GATCCGTAGGTGTCCTCTTCGGCAG |
|  | BamHI-*pqsA*-Prom-F2 | CGTAGGTGTCCTCTTCGGCAG |
|  | HindIII-*pqsA*-Prom-R1 | AGCTTGCGATATGCATCCGGATCAGG |
|  | HindIII-*pqsA*-Prom-R2 | TGCGATATGCATCCGGATCAGG |
| To construct pSB109-ArqI-BirA*-FLAG | *arqI-birA*-Link-R | CACCTGAACCACCACCACCACCCTCGACGCGGTAGC |
|  | *birA*-cterm-F | GGTGGTGGTGGTTCAGGTG |
|  | NdeI-*birA*-cterm-R1 | TATGTCATTTGTCGTCGTCGTCTTTGTAG |
|  | NdeI-*birA*-cterm-R2 | TGTCATTTGTCGTCGTCGTCTTTGTAG |
| To construct pSB109-BirA*-FLAG | NcoI-*birA*-cterm-F1 | CATGGGTGGTGGTGGTTCAGGTG |
|  | NcoI-*birA*-cterm-F2 | GGTGGTGGTGGTTCAGGT |
| Synthesized 5nm linker-BirA*-FLAG dsDNA fragment | 5nm linker-*birA**-FLAG | GGTGGTGGTGGTTCAGGTGGTGGTGGTTCAGGTGGTGGTGGTTCAATGTTCAAAAACCTTATCTGGCTTAAAGAAGTTGACTCAACTCAAGAACGTCTTAAAGAATGGAACGTTTCATACGGTACTGCTCTTGTTGCTGACCGTCAAACTAAAGGTCGTGGTGGTCTTGGTCGTAAATGGCTTTCACAAGAAGGTGGTCTTTACTTCTCATTCCTTCTTAACCCAAAAGAATTCGAAAACCTTCTTCAACTTCCACTTGTTCTTGGTCTTTCAGTTTCAGAAGCTCTTGAAGAAATCACTGAAATCCCATTCTCACTTAAATGGCCAAACGACGTTTACTTCCAAGAAAAAAAAGTTTCAGGTGTTTTATGCGAACTCAGCAAAGACAAACTTATCGTTGGTATCGGTATCAACGTTAACCAACGTGAAATCCCAGAAGAAATCAAAGACCGTGCTACTACTCTTTACGAAATCACTGGTAAAGACTGGGACCGTAAAGAAGTTCTTCTTAAAGTTCTTAAACGTATCTCAGAAAACCTTAAAAAATTCAAAGAAAAATCATTCAAAGAATTCAAAGGTAAAATCGAATCAAAAATGCTTTACCTTGGTGAAGAAGTTAAACTTCTTGGTGAAGGTAAAATCACTGGTAAACTTGTTGGTCTTTCAGAAAAAGGTGGTGCTCTTATCCTTACTGAAGAAGGTATCAAAGAAATCCTTTCAGGTGAATTCTCACTTCGTCGTTCAGACTACAAAGACGACGACGACAAATAA |
| To construct pCPD-PqsA | pCPD-*pqsA*-F | TTAACTTTAAGAAGGAGTCTCTCCCATGTCCACATTGGCCAACCTG |
|  | pCPD-*pqsA*-R | TTTTTCCATCCGCTAATGCTGCCCCACATGCCCGTTCCTCCGG |
| To construct pQLinkG2-PqsA-NTD | pQLinkG2-*pqsA*-NTD-F1 | GGCCATGTCCACATTGGCCAACCT |
|  | pQLinkG2-*pqsA*-NTD-F2 | ATGTCCACATTGGCCAACCT |
|  | pQLinkG2-*pqsA*-NTD-R1 | AGCTTCAGTCTTCCCGCCCACAGT |
|  | pQLinkG2-*pqsA*-NTD-R2 | TCAGTCTTCCCGCCCACAGT |
