# Supplementary material for: A glyoxal-specific aldehyde signaling axis in Pseudomonas aeruginosa that influences quorum sensing and infection
Source: Nat Commun. 2025 Jul 18;16:6616. doi: 10.1038/s41467-025-61469-8 (PMC12274486; doi:10.1038/s41467-025-61469-8)
Supplement: Supplementary file 8 — Reporting Summary [file 41467_2025_61469_MOESM8_ESM.pdf]

nature portfolio

Corresponding author(s): Andrew T. Ulijasz

Last updated by author(s): Apr 11, 2024

Reporting Summary

Nature Portfolio wishes to improve the reproducibility of the work that we publish. This form provides structure for consistency and transparency in reporting. For further information on Nature Portfolio policies, see our Editorial Policies and the Editorial Policy Checklist.

Statistics

For all statistical analyses, confirm that the following items are present in the figure legend, table legend, main text, or Methods section.

n/a	Confirmed	
☐	☒	The exact sample size (*n*) for each experimental group/condition, given as a discrete number and unit of measurement
☐	☒	A statement on whether measurements were taken from distinct samples or whether the same sample was measured repeatedly
☐	☒	The statistical test(s) used AND whether they are one- or two-sided<br>*Only common tests should be described solely by name; describe more complex techniques in the Methods section.*
☒	☐	A description of all covariates tested
☒	☐	A description of any assumptions or corrections, such as tests of normality and adjustment for multiple comparisons
☐	☒	A full description of the statistical parameters including central tendency (e.g. means) or other basic estimates (e.g. regression coefficient) AND variation (e.g. standard deviation) or associated estimates of uncertainty (e.g. confidence intervals)
☐	☒	For null hypothesis testing, the test statistic (e.g. *F*, *t*, *r*) with confidence intervals, effect sizes, degrees of freedom and *P* value noted<br>*Give P values as exact values whenever suitable.*
☒	☐	For Bayesian analysis, information on the choice of priors and Markov chain Monte Carlo settings
☒	☐	For hierarchical and complex designs, identification of the appropriate level for tests and full reporting of outcomes
☒	☐	Estimates of effect sizes (e.g. Cohen's *d*, Pearson's *r*), indicating how they were calculated

Our web collection on statistics for biologists contains articles on many of the points above.

Software and code

Policy information about availability of computer code

Data collection	Microscopy: Super resolution imaging data were collected using ZEN 2.3 SP1 FP3 (Zeiss, black edition, release version 14.0) with an Airyscan SR (acquisition) setting. For time-lapse imaging Nikon Elements Advanced Research software was used to acquire the data with a Ti2-E inverted microscope. FRAP images were acquired using custom microscopy control software (Morgenstein and Bratton et al., 2015 PNAS) written in LabView (National Instruments). LC-MS/MS: Mass spectrometry data was collected using FAIMS Pro interface running Tune 3.5 and Xcalibur 4.5. Crystal structures: Data were collected at the Stanford Synchrotron Radiation Lightsource (SSRL 12-2), or the Advanced Light Source at Berkley (native modified structure only; ALS 5.0.2). The SAD dataset was indexed and integrated using XDS (Kabsch et al. 2010), and scaled in AIMLESS (Evans, P.R. and G.N. Murshudov). EM: EM data were collected on a JEOL 3200FS transmission electron microscope (JEOL, Peabody, MA) operating at 300 keV with a Gatan K3 Direct Electron Detector (Gatan, Pleasanton, CA) using the program SerialEM version 3-8-9 (Mastronarde, D. N., 2015). A total of 1,054 movies were collected. Bioinformatics: Sequences of ABM domains were acquired from UniProt.
Data analysis	Microscopy: Data from the Airyscan microscope imaging was processed using a MicrobeJ Plugin version 5.13l (Ducret et al. 2015, Nature Microbiology) for ImageJ. Time lapse microscopy data were analyzed using ImageJ version 1.54f. FRAP data were processed in custom MATLAB code using multiple versions (2020a-2023b; MathWorks, Natwick, MA) and analyzed using custom MATLAB software (Konopka et al. 2006) from 20 imaged cells per strain on three separate days (biological repeats) for a total of 60 images per strain. These numbers were sufficient to yield statistically-significant data. LC-MS/MS: For P. aeruginosa data were searched against the PAO1 proteome using Peaks Bioinformatic software (Bioinformatics Solutions Inc). Data from the ArqI crystals were analyzed using Proteome Discover version 2.4 software. Data from E. coli purified ArqI was analyzed with Proteom Discoveror version 2.5 (Thermo Fisher) using Sequest HT search engines (Eng et al. 1994). Data has been depositied in MassIVE under the following link: MassIVE Private Dataset (ucsd.edu). Crystal structures: From the initial wild-type ArqI raw data phases were solved and a model was generated in Crank-2 in CCP4 (Pannu et al. 2011), and refined in phenix.refine version 1.20.1_4487 (Afonine et al. 2012) and Coot version 0.9.8.1 (Emsley et al. 2004). All subsequent datasets were solved by

molecular replacement in Phaser with phases supplied from the Se-SAD-derived ArqI dimer. The data from the native modified ArqI, unmodified ArqI, and R16A mutant have been deposited in the RCSB Protein Databank under PDB code numbers 8ECX, 8EIF, and 8ECP, respectively. EM: A total of 1,054 movies were collected. Data were processed and refined using RELION3 version 3.1.0 (Zivanov, J. et al. 2018). 57,667 particles were used to generate ab initio models. Bioinformatics: ArqI sequences were aligned using MAFFT v7.388 (Katoh et al. 2013) and tree files were generated using FastTree v2.1.11 (Price et al. 2009). iTOL was used for display (Letunic et al. 2007). Custom programing in BioPython and R were used to identify the Arg49 equivalent residue (deposited in GiTHub, https://github.com/putonti/stockholm_to_fasta). GraphPad Prism version 10.2.2 was used to calculate means, standard deviations, generate graphical data, and perform statistical analyses. Statistical tests where carefully applied depending on the sample size and experiment.

For manuscripts utilizing custom algorithms or software that are central to the research but not yet described in published literature, software must be made available to editors and reviewers. We strongly encourage code deposition in a community repository (e.g. GitHub). See the Nature Portfolio guidelines for submitting code & software for further information.

Data

Policy information about availability of data

All manuscripts must include a data availability statement. This statement should provide the following information, where applicable:

- Accession codes, unique identifiers, or web links for publicly available datasets
- A description of any restrictions on data availability
- For clinical datasets or third party data, please ensure that the statement adheres to our policy

If not already contained within the manuscript files, upon request, raw data are available from the authors for all experiments. The data from the native modified ArqI, unmodified ArqI, and R16A mutant have been deposited in the RCSB Protein Databank under PDB code numbers 8ECX, 8EIF, and 8ECP, respectively.

Research involving human participants, their data, or biological material

Policy information about studies with human participants or human data. See also policy information about sex, gender (identity/presentation), and sexual orientation and race, ethnicity and racism.

Reporting on sex and gender	*Use the terms sex (biological attribute) and gender (shaped by social and cultural circumstances) carefully in order to avoid confusing both terms. Indicate if findings apply to only one sex or gender; describe whether sex and gender were considered in study design; whether sex and/or gender was determined based on self-reporting or assigned and methods used.*<br>*Provide in the source data disaggregated sex and gender data, where this information has been collected, and if consent has been obtained for sharing of individual-level data; provide overall numbers in this Reporting Summary. Please state if this information has not been collected.*<br>*Report sex- and gender-based analyses where performed, justify reasons for lack of sex- and gender-based analysis.*
Reporting on race, ethnicity, or other socially relevant groupings	*Please specify the socially constructed or socially relevant categorization variable(s) used in your manuscript and explain why they were used. Please note that such variables should not be used as proxies for other socially constructed/relevant variables (for example, race or ethnicity should not be used as a proxy for socioeconomic status).*<br>*Provide clear definitions of the relevant terms used, how they were provided (by the participants/respondents, the researchers, or third parties), and the method(s) used to classify people into the different categories (e.g. self-report, census or administrative data, social media data, etc.)*<br>*Please provide details about how you controlled for confounding variables in your analyses.*
Population characteristics	*Describe the covariate-relevant population characteristics of the human research participants (e.g. age, genotypic information, past and current diagnosis and treatment categories). If you filled out the behavioural & social sciences study design questions and have nothing to add here, write "See above."*
Recruitment	*Describe how participants were recruited. Outline any potential self-selection bias or other biases that may be present and how these are likely to impact results.*
Ethics oversight	*Identify the organization(s) that approved the study protocol.*

Note that full information on the approval of the study protocol must also be provided in the manuscript.

Field-specific reporting

Please select the one below that is the best fit for your research. If you are not sure, read the appropriate sections before making your selection.

☒ Life sciences ☐ Behavioural & social sciences ☐ Ecological, evolutionary & environmental sciences

For a reference copy of the document with all sections, see nature.com/documents/nr-reporting-summary-flat.pdf

Life sciences study design

All studies must disclose on these points even when the disclosure is negative.

Sample size	No sample size calculation was made, or statistical method applied to assess sample size, save for number of mice selected for performing the sepsis infections (see below). For quantification of ArqI-sfGFP foci, a range of 50-486 bacterial cells were analyzed per biological replicate. For generation of fluorescence intensity heatmaps, a minimum of 250 cells were utilized. These numbers are typical of bacterial microscopy and sufficient to yield statistically significant data between strains/sample types. For murine sepsis infection experiments, a total of 10 mice per strain gives sufficient statistical power allowing for the usual variability we have seen with P. aeruginosa sepsis infections (Allen et al. 2020, PNAS; PMID 32156726). For EM, 57,667 particles were chosen, which is typical for what our negative EM experiments required for a 3D structure (Glanville et al. 2023, PLoS Pathogens).
Data exclusions	For ArqI-sfGFP quantification and heatmap generation cells less than 1 μM in length and at the edge of images were excluded to prevent cells not lying flat in the focal plane, or not fully in the frame from being analyzed. For EM images, successive rounds of 2D classification were used to remove noise and bad particles yielding 57,667 final particles, which were used to generate ab initio models and in all subsequent steps.
Replication	When possible, technical replicates were performed in addition to (at least 3) biological replicates. Most often, biological replicates were performed on different days. RNA-Seq experiments were performed in biological triplicate, an 'n' value of which has been determined to give sufficient statistics/p-values between samples (Li D, Zand MS, Dye TD, Goniewicz ML, Rahman I, Xie Z (2022) An evaluation of RNA-seq differential analysis methods. PLoS ONE 17(9):e0264246. Significance (p-values of < 0.05) could be assigned to many genes with only a 2-fold difference between samples. In general, for bacterial culture from well-characterized bacterial strains, the expectation of reproducibility is high and three experimental replicates is expected to be adequate for determination of statistical significance. In our specific data here, the expectation is supported by the whisker-plot of non-normalized data (available upon request), in which the total average expression and dynamic range of expression is highly similar across both experimental treatments and biological replicates. Where appropriate, in the paper figure legends we give the number of replicates for each experiment. Glyoxal toxicity plate assays were performed in biological triplicate. Fluorescence reporter assays were performed in technical and biological triplicate. Size exclusion chromatography (SEC) was performed twice resulting in the same peak profiles both times. For foci quantification and heatmap generation, three representative fields per biological replicate (in minimum triplicate) were utilized for analyses. Time lapse imaging was performed in biological triplicate. FRAP analysis was performed in biological triplicate. Pyocyanin and pyoverdine measurements were performed in technical triplicate and biological quadruplet. Extraction of PQS and TLC was performed in biological quadruplet. Intrinsic tryptophan quenching was performed in biological quadruplet. Western blots were performed at a minimum of two times on different days. Sepsis infections were performed on two separate days, with 5 mice per strain, per day with a total of 10 mice per strain.
Randomization	n/a
Blinding	n/a

Reporting for specific materials, systems and methods

We require information from authors about some types of materials, experimental systems and methods used in many studies. Here, indicate whether each material, system or method listed is relevant to your study. If you are not sure if a list item applies to your research, read the appropriate section before selecting a response.

Materials & experimental systems

n/a	Involved in the study	
☐	☒	Antibodies
☒	☐	Eukaryotic cell lines
☒	☐	Palaeontology and archaeology
☐	☒	Animals and other organisms
☒	☐	Clinical data
☒	☐	Dual use research of concern
☒	☐	Plants

Methods

n/a	Involved in the study	
☒	☐	ChIP-seq
☒	☐	Flow cytometry
☒	☐	MRI-based neuroimaging

Antibodies

Antibodies used	The following antibodies were used in these studies: Mouse anti-FLAG (Clone M2, Cat# F3165, Lot SLCC4005, Sigma Aldrich), rabbit anti-HA (Polyclonal, Cat# H6908, Sigma), rabbit anti-GFP (Polyclonal, Cat# A11122, Lot 2180255, Invitrogen), mouse anti-RpoB (Clone 8RB13, Cat# 663903, Lot B287124, Biolegend, San Diego, CA), horseradish peroxidase (HRP) -conjugated goat anti-mouse (Cat# 115-035-003, Lot 130388, Jackson ImmunoResearch, UK), HRP-conjugated goat anti-rabbit (Cat# 111-035-003, Lot 131402, Jackson ImmunoResearch), Streptavidin-HRP (Cat# AB7403, Lot GR3368592-7, Abcam), Mouse anti-HA (Clone 16B12, Cat# 901515, Lot B355626, Biolegend).

Validation	Mouse anti-FLAG M2, rabbit anti-HA, and mouse anti-RpoB used against Pseudomonas aeruginosa cell extracts have been published previously; Trouillon et al. 2022 (Nature Communications), Imbert et al. 2017 (The EMBO Journal), and Kung et al. 2012 (PNAS), respectively. Rabbit anti-GFP used against E. coli cell extracts has been published previously; Hunt et al. 2021 (Pathogens and Disease). Streptavidin-HRP has been used previously with Mycobacterium smegmatis; (Veyron-Churlet et al. 2023 (FASEB Journal). Mouse anti-HA against P. aeruginosa cell extracts has been published previously by Imbert et al. 2017 (The EMBO Journal).

Animals and other research organisms

Policy information about studies involving animals; ARRIVE guidelines recommended for reporting animal research, and Sex and Gender in Research

Laboratory animals	Female BALB/c mice aged 6 to 8 weeks, purchased from The Jackson Laboratory.
Wild animals	Wild animals were not used in this study.
Reporting on sex	Only female mice were used in the study, and sex was not considered in the current study design. Prior work using this infection model has demonstrated no sex-dependent differences in phenotypes. PMIDs 32156726, 31992714.
Field-collected samples	There were no field-collected samples in this study.
Ethics oversight	Studies were approved by the Institutional Animal Care and Use Committee (IACUC) at Loyola University Chicago (Protocol 2021016).

Note that full information on the approval of the study protocol must also be provided in the manuscript.

Plants

Seed stocks	*Report on the source of all seed stocks or other plant material used. If applicable, state the seed stock centre and catalogue number. If plant specimens were collected from the field, describe the collection location, date and sampling procedures.*
Novel plant genotypes	*Describe the methods by which all novel plant genotypes were produced. This includes those generated by transgenic approaches, gene editing, chemical/radiation-based mutagenesis and hybridization. For transgenic lines, describe the transformation method, the number of independent lines analyzed and the generation upon which experiments were performed. For gene-edited lines, describe the editor used, the endogenous sequence targeted for editing, the targeting guide RNA sequence (if applicable) and how the editor was applied.*
Authentication	*Describe any authentication procedures for each seed stock used or novel genotype generated. Describe any experiments used to assess the effect of a mutation and, where applicable, how potential secondary effects (e.g. second site T-DNA insertions, mosiacism, off-target gene editing) were examined.*
